# Polymerizable Zwitterionic Liquid Stationary Phases With Improved Thermal Stability for Separation of Carboxylic Acids

**DOI:** 10.1002/jssc.70496

**Published:** 2026-07-22

**Authors:** Bhawana Thapa, Jessica F. DeLair, Jared L. Anderson

**Affiliations:** ^1^ Department of Chemistry Iowa State University Ames Iowa USA

**Keywords:** carboxylic acid separation, gas chromatography, thermal stability, zwitterionic liquid stationary phase

## Abstract

Polymerizable zwitterionic liquids (ZILs) were investigated for the development of thermally stable wall‐coated open‐tubular (WCOT) stationary phases for gas chromatography (GC) in the separation of volatile carboxylic acids (VCAs). Three imidazolium‐based ZILs containing alkenyl and acrylate functional groups were synthesized and evaluated based on their polymerization and used as separation media. Among the evaluated chemical structures, only the acrylate ZIL demonstrated the ability to undergo free radical polymerization and was subsequently employed in the preparation of chromatographic columns. WCOT columns were prepared by the static coating method followed by in‐situ polymerization inside the GC capillary columns, resulting in chromatographic efficiencies up to 3300 plates m^−1^. The resulting stationary phase enabled separation of VCAs under isothermal conditions at 100°C and exhibited reduced bleed (from 6.92–7.80 pA at 40°C to 154.70–157.57 pA at 250°C) compared to a corresponding non‐polymerizable ZIL (from 7.58 pA at 40°C to 396.38 pA at 250°C), indicating improved thermal stability of the stationary phase upon polymerization. However, thermal treatment up to 250°C resulted in no chromatographic retention of benzyl alcohol, suggesting that the stationary phase undergoes an increase in rigidity under these conditions. To address this limitation, poly(ethylene glycol) methacrylate (PEGMA) was incorporated as a co‐monomer at 10%, 20%, 25%, and 40% weight fractions to introduce flexibility within the polymeric network. With increasing PEGMA content, progressively narrower peaks of benzyl alcohol were observed after thermal treatment (3.3, 1.10, 0.81, and 0.194 min for 10%, 20%, 25%, and 40% weight fractions, respectively), indicating improved mass transfer within the stationary phase. These results demonstrate the feasibility of polymerizable ZIL stationary phases for GC and provide insight into the role of polymer composition in controlling chromatographic performance after thermal treatment.

## Graphical abstract

Polymerizable acrylate zwitterionic liquid stationary phases provide improved thermal stability and reduced column bleed for gas chromatography while maintaining effective separation of volatile carboxylic acids. Incorporation of poly(ethylene glycol) methacrylate enhances polymer flexibility and improves mass transfer following thermal exposure.

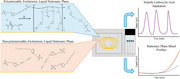

## 1 Introduction

Highly polar acidic compounds such as volatile carboxylic acids (VCAs) and free fatty acids are often difficult to analyze by gas chromatography (GC) in their free acid form due to their strong intermolecular interactions and tendency to interact with residual silanol groups or other active sites present on the surface of open tubular (OT) capillary columns [1, 2]. These interactions can lead to peak tailing, distorted peak shapes, and reduced quantitative accuracy, as peak area integration can be affected with tailing peaks. In severe cases, irreversible adsorption of analytes may occur, preventing their complete elution from the column [3]. Accurate determination of these compounds is highly important as they are widely used in the production of fuels, cosmetics, pharmaceuticals, and food, while serving as indicators of food quality [4, 5, 6, 7]. Derivatization is frequently employed prior to GC analysis to convert carboxylic acids into less polar and more volatile derivatives [8, 9]. Although derivatization can improve chromatographic peak shapes and volatility, it often introduces additional steps into the analytical workflow and may generate additional complications that must be addressed. Derivatized samples may contain excess derivatizing reagents, incompletely derivatized analytes, and various reaction by‐products, which can make interpretation of chromatographic data challenging [10, 11]. Consequently, the development of stationary phases capable of directly separating acidic compounds without derivatization remains an important objective in GC analysis.

The range of commercially available polar stationary phases for GC separations remains relatively limited. Polyethylene glycol (PEG)‐based stationary phases constitute one of the most widely used classes of polar GC stationary phases. Among these, acid‐modified PEG phases are designed primarily for the analysis of free fatty acids [12, 13]. However, PEG‐based stationary phases typically exhibit lower thermal stability, with maximum operating temperatures around 260°C. This limitation has motivated continued exploration of alternative stationary phase chemistries capable of providing stronger and more selective interactions with polar analytes while maintaining adequate thermal robustness.

Ionic liquids (ILs) have emerged as promising stationary phases in GC due to their negligible vapor pressure, high thermal stability, and tunable chemical structures [14, 15]. By modifying the cationic and anionic components of these materials, IL‐based stationary phases can provide diverse intermolecular interactions, including dispersive, dipolar, and hydrogen bonding interactions [16]. Among these materials, a subclass of ILs called zwitterionic liquids (ZILs) represents a particularly attractive class of stationary phases, as both the cationic and anionic functional groups are tethered within the same molecular structure. The arrangement of the oppositely charged ions imparts ZILs with exceptional hydrophilicity while maintaining overall charge neutrality, making them well‐suited for the separation of highly polar compounds [17, 18].

ZILs were first reported as GC stationary phases in the separation of VCAs in 2019, where the strong retention of acidic analytes was attributed largely to the high hydrogen bond basicity of the anionic component [17]. The ZIL stationary phases exhibited higher retention factors and improved peak asymmetry compared to commercial polar columns used for fatty acid analysis. In particular, alkyl‐substituted ZILs were shown to provide higher and more selective retention of less polar VCAs compared with oligoether‐functionalized ZILs and a commercial Stabilwax column, indicating that analyte retention is largely governed by the nature of the cation substituents. However, ZIL stationary phases incorporating oligoether‐functionalized substituents have been shown to provide a more suitable compromise between VCA retention, peak shape, and thermal stability. Despite their favorable chromatographic performance, the initial ZIL stationary phases exhibited relatively limited thermal stability, with maximum allowable operating temperatures between approximately 200 and 225°C. While zwitterionic stationary phases can provide enhanced selectivity for acidic analytes, further improvements in the overall thermal stability are necessary to expand their practical applicability in GC. Subsequent investigations have aimed at examining the thermal behavior of imidazolium‐based ZILs, which revealed that both the cationic substituents and anionic components play important roles in their overall thermal stability [18, 19]. Thermal degradation studies of imidazolium sulfonate ZILs suggested that degradation commonly occurs through nucleophilic attack of the sulfonate anion on the α‐carbon of the imidazolium ring. Modification of the ZIL structure, including substitution of the sulfonate anion with more thermally stable anions, such as triflimide (NTf), has been shown to improve thermal stability and alter the thermal degradation pathways of these ZILs; however, this modification of the ZIL resulted in poor retention and increased peak asymmetry of the VCAs. In addition to chemical degradation, the ability of ZIL stationary phases to maintain a stable thin film on the capillary surface is also critical for chromatographic performance. A previous study reported loss of column efficiency and decreased analyte retention after extended heating, indicating phase rearrangement or partial loss of the stationary phase at elevated temperatures [18].

The aforementioned studies have collectively demonstrated that while ZIL stationary phases offer promising selectivity for the separation of acidic analytes, further improvements in thermal and film stability are required to fully realize their potential. One promising strategy to address these challenges is the incorporation of polymerizable functional groups into the ZIL chemical structure. Polymerizable ILs have been covalently immobilized or assist in the formation of crosslinked networks within the GC capillary, to improve resistance to thermal degradation and reduce stationary phase bleed during high temperature operation [20, 21]. In this work, three ZIL chemical structures containing polymerizable moieties were designed, synthesized, and evaluated for their potential to undergo polymerization. A propanesulfonate group was selected as the anionic moiety because of its reported ability to promote favorable interactions with VCAs, while alkyl (octenyl) and oligoether (vinylPEG‐2) substituents bearing terminal alkene functionalities were incorporated to investigate the influence of cation functionality and facilitate covalent immobilization of the stationary phase. To further examine the effect of the polymerizable moiety, a third ZIL containing an acrylate group instead of a terminal alkene was also designed. The ability of these compounds to polymerize was first examined outside the column under controlled conditions to assess the stability and reactivity of the polymerizable functional groups prior to their subsequent evaluation within the GC capillary column. Thermal stability was assessed by constructing bleed profiles and by comparison with a non‐polymerizable ZIL and a cross‐bonded commercial stationary phase. Additionally, the influence of a co‐monomer on the rigidity of the resulting polymeric stationary phase was investigated.

## 2 Experimental

### 2.1 Materials and Reagents

The synthesis of ZIL stationary phases utilized commercially available reagents. Triethylamine (≥99.5%), 1,3‐propanesultone (98%), 1H‐imidazole (99%), and acrylonitrile (≥99%) were obtained from Aldrich Chemical Company. Methanesulfonyl chloride (98%) and sodium hydroxide (98%) were supplied by Thermo Scientific (Waltham, MA, USA). Potassium hydroxide (>90%) and di(ethylene glycol) vinyl ether (VinylPEG‐2, 98%) were purchased from Sigma Aldrich (St. Louis, MO, USA). The reagent 6‐bromohexylacrylate (95%, stabilized with MEHQ) was purchased from Ambeed Inc. (Arlington Heights, IL, USA), and 8‐bromo‐1‐octene (>98%) was purchased from Tokyo Chemical Industry (Tokyo, Japan). Organic solvents used during synthesis and purification, including dichloromethane (≥99.5%), acetone (≥99.5%), and acetonitrile (≥99.95%), were purchased from Fisher Scientific (Waltham, MA, USA), with hexanes (≥98.5%), ethyl acetate (≥99.5%), and methanol (≥99.9%) being purchased from Sigma Aldrich. Deuterated solvents used for nuclear magnetic resonance (NMR) analysis included chloroform‐d and deuterium oxide (D_2_O) obtained from Sigma Aldrich and dimethyl sulfoxide‐d_6_ from Cambridge Isotope Laboratories (Tewksbury, MA, USA).

VCA standards used in this study were acetic acid (≥99%) purchased from Fisher Scientific, propionic acid (∼99%), butyric acid (≥99%), and valeric acid (≥99%) from Sigma‐Aldrich (St. Louis, MO, USA), and hexanoic acid (98+%) from Thermo Fisher Scientific. Individual VCA standard solutions were prepared in acetonitrile at concentrations of 1000 mg L^−^^1^. Benzyl alcohol (≥99%, Sigma Aldrich) was used as a probe compound for column efficiency measurements and prepared as a 1000 mg L^−^^1^ solution in acetonitrile. Untreated fused silica capillary tubing (60 m × 0.25 mm i.d.) used for the fabrication of GC columns and the commercial column Stabilwax (30 m × 0.25 mm i.d × 0.25 µm d_f_) were obtained from Restek Corporation (Bellefonte, PA, USA), and PTFE tubing (0.012 ± 0.001 in. i.d.) used during column preparation was purchased from Zeus Industrial Products (Orangeburg, SC, USA). Ultra‐high purity helium, ultra‐high purity hydrogen, and Grade D breathing air used for operation of the GC–FID instrument were supplied by Matheson Tri‐Gas (Irving, TX, USA).

### 2.2 Synthesis of ZILs

The detailed syntheses of all ZILs employed in this study can be found in the Supporting Information, with synthetic schematics for Vinyl‐PEG2‐ImC_3_SO_3_ and the acrylate ZIL shown in Figures S1 and S2, respectively. The purities of all ZILs were confirmed by ^1^H NMR and ^13^C NMR.

### 2.3 Preparation and Testing of Coated Column With ZIL Stationary Phase

Wall‐coated OT (WCOT) columns were prepared using the static coating method. Coating solutions were prepared by dissolving 0.40% (w/v) ZIL in a solvent mixture consisting of 8% (v/v) methanol in dichloromethane to obtain a stationary phase film thickness of 0.25 µm. Azobisisobutyronitrile (AIBN) was added to the coating solution as a free radical initiator at 3% (w/w) relative to the ZIL mass. For PEGMA‐containing columns, the relative masses of ZIL and PEGMA were adjusted such that the final stationary phase film thickness remained approximately 0.25 µm. All 5 m capillaries were coated at 45°C under a vacuum of 23 inHg. Vinyl trimethoxy silane (VTMS) modified capillary tubing was prepared according to a previously reported procedure [20] and subsequently coated using the same static coating method.

After the coating process, all columns were sealed and subjected to polymerization by heating within a GC oven from 40°C to 80°C at 2°C min ^−1^ and then held isothermally at 80°C for 15 h [20, 21]. Following polymerization, the columns were conditioned at 100°C for 2–3 h until the baseline stabilized prior to efficiency evaluation. To test column efficiency, 1 µL of a 1000 µg mL^−1^ standard solution of benzyl alcohol in ACN was injected with a 20:1 split ratio and the separation carried out under the isothermal condition at 100°C. Bleed profiles for the columns were obtained by increasing the oven temperature from 40°C to 250°C at a ramp rate of 0.5°C min^−1^.

### 2.4 Instrumentation

The purity of all synthesized ZILs was evaluated using NMR spectroscopy. Proton (^1^H) and carbon‐13 (^13^C) NMR spectra were acquired using a Bruker Avance Neo 400 spectrometer and a Varian MR 400 spectrometer. Column efficiency, retention measurements, and bleed profile analyses were performed using an Agilent 7890 gas chromatograph equipped with a flame ionization detector (GC–FID). The FID signal was recorded in picoamperes (pA). The GC was operated using helium as the carrier gas at a flow rate of 1 mL min^−^^1^, with the inlet temperature maintained at 250°C. The FID temperature was maintained at 250°C, with air, hydrogen (fuel), and makeup gas flow rates of 395, 35, and 25 mL min^−^^1^, respectively. Fourier‐transform infrared (FTIR) spectra were recorded over the wavenumber range of 400–4000 cm^−^^1^, with each spectrum obtained by averaging 64 scans at a resolution of 4 cm^−^^1^.

## 3 Results and Discussion

### 3.1 Polymerization and Characterization of ZILs Prepared Outside the Chromatographic Column

**FIGURE 1:**
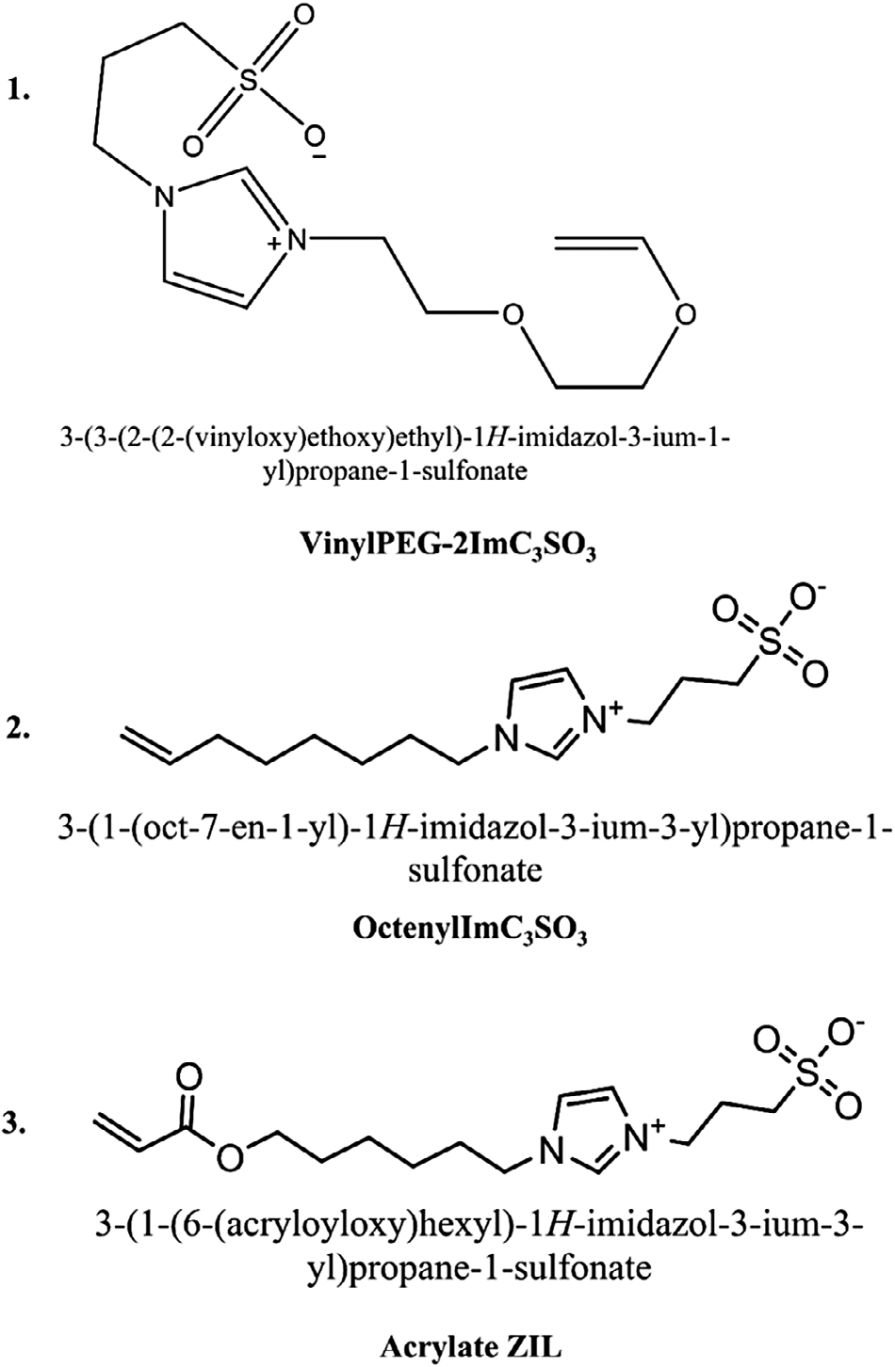
Chemical structures, full names, and abbreviations of the zwitterionic liquids synthesized and evaluated as gas chromatography (GC) stationary phases in this study.

Before attempting polymerization of the ZILs inside the GC capillary column, polymerization of all synthesized ZILs (Figure 1) was first evaluated outside of the column. Polymerization experiments were performed either by heating the ZILs in a vacuum oven or by carrying out the reaction in a round‐bottom flask in the presence of AIBN as a free radical initiator. These preliminary experiments were carried out to confirm the propensity of the ZILs to undergo polymerization prior to their immobilization within the GC columns. The resulting materials were subsequently characterized by ^1^H NMR, and samples that were successively polymerized were further analyzed by FTIR spectroscopy to confirm polymer formation.

The ZIL containing the VinylPEG‐2 moiety was evaluated first to understand its stability and polymerization behavior before attempting polymerization within the GC capillary. During these experiments, it was observed that the vinyl functionality was readily lost during storage or drying. When the synthesized ZIL was dried using rotary evaporation followed by storage in a desiccator or by placing the material in a room temperature vacuum oven, the vinyl proton signals that initially appeared in the 4–6.5 ppm region (Figure S3) were no longer detected in the ^1^H NMR spectra. Instead, new peaks appeared and were observed to be dependent on the storage conditions. Samples stored in a desiccator exhibited a new peak at approximately 3.21 ppm (Figure S4), whereas samples dried in a vacuum oven showed the appearance of a peak near 1.26 ppm (Figure S5). Despite these changes, the material remained similar in appearance to the original product and retained solubility in the same organic solvents as the monomer, including common NMR solvents. In addition, the NMR spectra did not exhibit peak broadening typically associated with polymer formation, suggesting that polymerization had not occurred [22, 23]. These observations indicate that the vinyl functionality in this ZIL may be prone to chemical transformation under storage conditions, which may be related to the presence of the sulfonate group, as the corresponding intermediate lacking the sulfonate functionality remained stable under similar drying and storage conditions. Therefore, this compound was considered unsuitable for subsequent immobilization within VTMS‐modified GC capillary columns.

The ZIL containing an octenyl moiety was evaluated similarly, in which the synthesized ZIL (^1^H NMR shown in Figure S6) was first placed overnight in a heated vacuum oven at 60°C to determine whether polymerization could be initiated thermally. The ^1^H NMR spectrum obtained the following day showed no observable changes, indicating that the compound remained unchanged and that heat alone did not appear to initiate polymerization. Subsequently, a polymerization experiment was conducted in a round‐bottom flask using 3% (w/w) AIBN as a free radical initiator in ACN. The reaction mixture was purged with N_2_ and heated at 80°C. After 24 h, the ^1^H NMR spectrum showed no significant changes, with signals from the vinyl proton remaining intact. An additional 3% AIBN was added, and the system was again purged with N_2_, and the reaction allowed to proceed overnight. However, the subsequent NMR spectrum again showed the presence of vinyl peaks with no appearance of new peaks or peak broadening. These observations indicated that the octenyl functionalized ZIL was not amenable to undergoing free radical polymerization under the conditions employed. This behavior is consistent with reports that α‐olefins (terminal alkenes) are difficult to homopolymerize under conventional free‐radical conditions, as propagating radicals preferentially undergo allylic hydrogen abstraction, generating stable allylic radicals that inhibit chain growth [24, 25]. Although radical polymerization of simple terminal alkenes has been reported under specialized catalytic systems, such as Li^+^‐catalyzed radical polymerization [25, 26], the use of such catalysts inside GC capillaries could introduce additional complications related to catalyst compatibility and column preparation. Therefore, this approach was not pursued in the present study.

**FIGURE 2:**
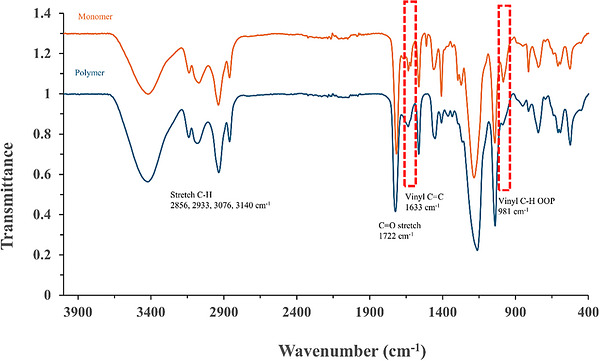
Fourier‐transform infrared spectrum of the acylate zwitterionic liquid monomer and zwitterionic liquid polymer, indicating the disappearance of the vinyl group.

Because the VinylPEG‐2ImC_3_SO_3_ ZIL showed instability of the vinyl functional group and the octenyl functionalized ZIL did not undergo free radical polymerization, a third ZIL containing acrylate functionality was synthesized and evaluated. The ^1^H and ^13^C NMR spectra of the acrylate ZIL are shown in Figures S7 and S8, respectively. The synthesized acrylate ZIL was placed in a glass vial and heated in a vacuum oven overnight at 60°C to study its ability to undergo polymerization. After heating, the initially viscous liquid material was transformed into a hard solid that strongly adhered to the walls of the glass vial, making it difficult to remove, as shown in Figure S9. The material was removed from the vial and washed with organic solvents, after which it exhibited a rubbery consistency and remained insoluble in common organic solvents, indicative that a polymeric material was formed. The recovered polymer was subsequently analyzed by FTIR spectroscopy, which revealed a significant reduction and broadening of the C = C stretching band (Figure 2), consistent with transformation of the double bond during polymerization [22, 23]. These observations confirmed that the acrylate functionalized ZIL readily undergoes polymerization upon application of heat and demonstrate that it has compatibility for in‐situ polymerization within GC capillary columns.

### 3.2 Chromatographic Performance and Thermal Behavior of the Polymerized ZIL Stationary Phase

The acrylate ZIL was coated onto both untreated and VTMS‐modified GC capillaries and subsequently subjected to polymerization and conditioning, as described in Section 2.3. The chromatographic performance of the resulting columns was then evaluated to assess the suitability of the polymerized GC stationary phase. Column efficiency was measured using benzyl alcohol as a probe molecule at 100°C. For the ZIL on the untreated capillary column, a separation efficiency of approximately 1900 plates m^−^^1^ was measured, while ZIL on the VTMS modified capillary showed a much lower efficiency of approximately 100 plates m^−1^, resulting from broader chromatographic peaks, as shown in Table 1. This behavior may be attributed to differences in surface polarity between the relatively less polar VTMS‐modified surface and the polar ZIL stationary phase, which can influence the distribution of the stationary phase within the capillary and affect mass transfer characteristics. In initial testing, four VCAs (propionic acid, butyric acid, valeric acid, and hexanoic acid) were used to examine the separation capability of the polymerized stationary phase. Overlayed chromatograms obtained from the untreated and VTMS modified capillaries, shown in Figure S10, demonstrated that the polymerized ZIL on untreated capillary was capable of separating the VCAs with good peak asymmetry values of <1.2 (Table S1) while the VTMS modified capillary could not effectively separate VCAs (Table S2), as the peaks were very broad (ranging from approximately 0.4–1.1 min, as shown in Table S2) and ultimately affected their resolution.

**TABLE 1:** Retention factor, column efficiency, and peak width at half height at 100°C measured after polymerization and conditioning (see section 2.3) for all prepared columns. The commercial Stabilwax column was measured following conditioning. Benzyl alcohol was used as a probe molecule in all chromatographic separations, and all columns were 5 m × 0.25 mm I.D.

Stationary phase	Retention factor^a^ ± SD	Efficiency (plates m^−1^)^b^ ± SD	Peak width at half height
100% acylate ZIL	42.7 ± 0.3	1881 ± 56	0.26 ± 0.01
100% acylate ZIL^c^	30.63 ± 0.03	101 ± 5	0.81 ± 0.02
Acrylate ZIL + 10% PEGMA ^d^	48.0 ± 0.7	2140 ± 93	0.289 ± 0.002
Acrylate ZIL + 20% PEGMA	50.1 ± 0.4	2945 ± 149	0.26 ± 0.01
Acrylate ZIL + 25% PEGMA	56.4 ± 0.4	3147 ± 40	0.280 ± 0.002
Acrylate ZIL + 40% PEGMA	44.5 ± 0.1	3303 ± 60	0.221 ± 0.003
100% PEGMA	27.8 ± 0.2	402 ± 9	0.40 ± 0.01
Stabilwax	33.20 ± 0.01	3223 ± 5	0.1638 ± 0.0002

^a^ Calculated using propane as a dead time marker.

^b^ Determined using the width at half‐height of the chromatographic peak (benzyl alcohol).

^c^ Coated on a vinyl trimethoxy silane modified surface. All other columns were coated on an untreated capillary.

^d^ ZIL: zwitterionic liquid, PEGMA: poly(ethylene glycol) methacrylate.

SD: Standard deviation calculated for retention factor and efficiency with *n* = 3 measurements.

**FIGURE 3:**
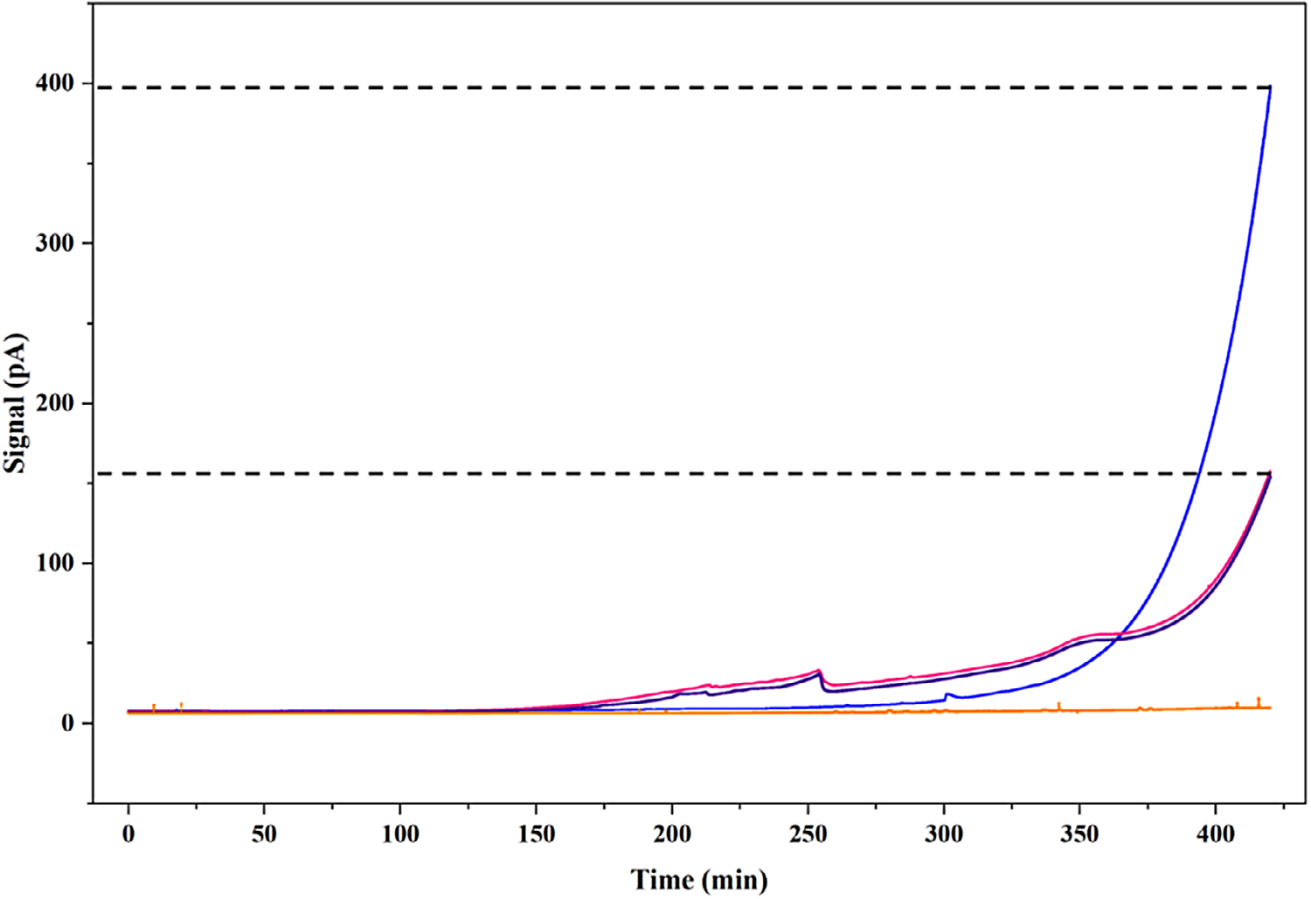
Comparison of the bleed profile of C_8_ImC_3_SO_3_ (non‐polymerizable zwitterionic liquid) (blue), acrylate zwitterionic liquid on untreated (purple) and vinyl trimethoxy silane modified capillary (pink), and commercial Stabilwax column (orange).

To evaluate the thermal stability of the polymerized stationary phase, bleed profiles were investigated by gradually exposing the columns to increasing temperatures up to 250°C, as described in Section 2.3. The resulting bleed traces are shown in Figure 3. For reference comparisons, the bleed profiles of a previously reported non‐polymerizable ZIL phase (C_8_ImC_3_SO_3_) and a cross‐bonded commercial stationary phase, Stabilwax, are also included. The polymerized acrylate‐based ZIL columns exhibited substantially lower bleed intensity (from 7.80 pA at 40°C to 157.57 pA at 250°C on VTMS‐modified capillary and from 6.92 pA at 40°C to 154.70 pA at 250°C on untreated capillary), approximately half that observed for the non‐polymerizable ZIL (from 7.58 pA at 40°C to 396.38 pA at 250°C), indicating improved thermal stability gained from polymerization of the ZIL stationary phase. Notably, the bleed profiles obtained for the untreated and VTMS‐modified capillaries were nearly identical. The bleed profile of Stabilwax, as shown in Figure 3, remained considerably consistent throughout the thermal treatment (from 6.57 pA at 40°C to 9.97 pA at 250°C), indicating that while the polymerized acrylate ZIL has improved thermal stability compared to a non‐polymerizable ZIL, further optimization is needed to achieve thermal stability comparable to that of commercial stationary phases.

Following exposure of the columns to elevated temperatures, benzyl alcohol was injected again at 100°C to reassess chromatographic performance. Under these conditions, benzyl alcohol eluted near the solvent front on both acrylate ZIL columns, indicating a loss of chromatographic retention. However, optical microscopy images confirmed the presence of the stationary phase within the capillary column (Figure S11), suggesting that the ZIL had not been removed from the column. These observations suggest that exposure to higher temperatures likely resulted in the polymerized stationary phase becoming highly rigid or more plastic‐like, thereby limiting mass transfer into the stationary phase and reducing chromatographic retention. While separation of VCAs could still be achieved efficiently at lower operating temperatures, efforts were undertaken to reduce the rigidity of the polymerized phase to improve its chromatographic performance after exposure to elevated temperatures.

### 3.3 Effect of Co‐Monomer on Reducing Polymer Rigidity and Improving Chromatographic Performance

In polymer systems, plasticizers are commonly used to improve the flexibility and reduce the rigidity of polymer networks. Plasticization can occur through the incorporation of external plasticizers, which are physically blended with the polymer matrix, or through internal plasticizers, which are chemically incorporated into the polymer structure during polymerization [27]. Internal plasticization introduces flexible segments within the polymeric network, thereby increasing chain mobility and reducing intermolecular interactions between polymer chains. In methacrylate‐based polymer systems, poly(ethylene glycol) methacrylate (PEGMA) and related oligo (ethylene glycol) methacrylates are widely used as co‐monomers because their methacrylate functionality allows co‐polymerization, while the flexible PEG side chains introduce mobility within the polymer structure and influence overall thermal and phase behavior [28, 29]. In the present study, PEGMA was incorporated as a co‐monomer during the polymerization of the ZIL stationary phase to attempt to reduce the rigidity observed after exposure to elevated temperatures.

**FIGURE 4:**
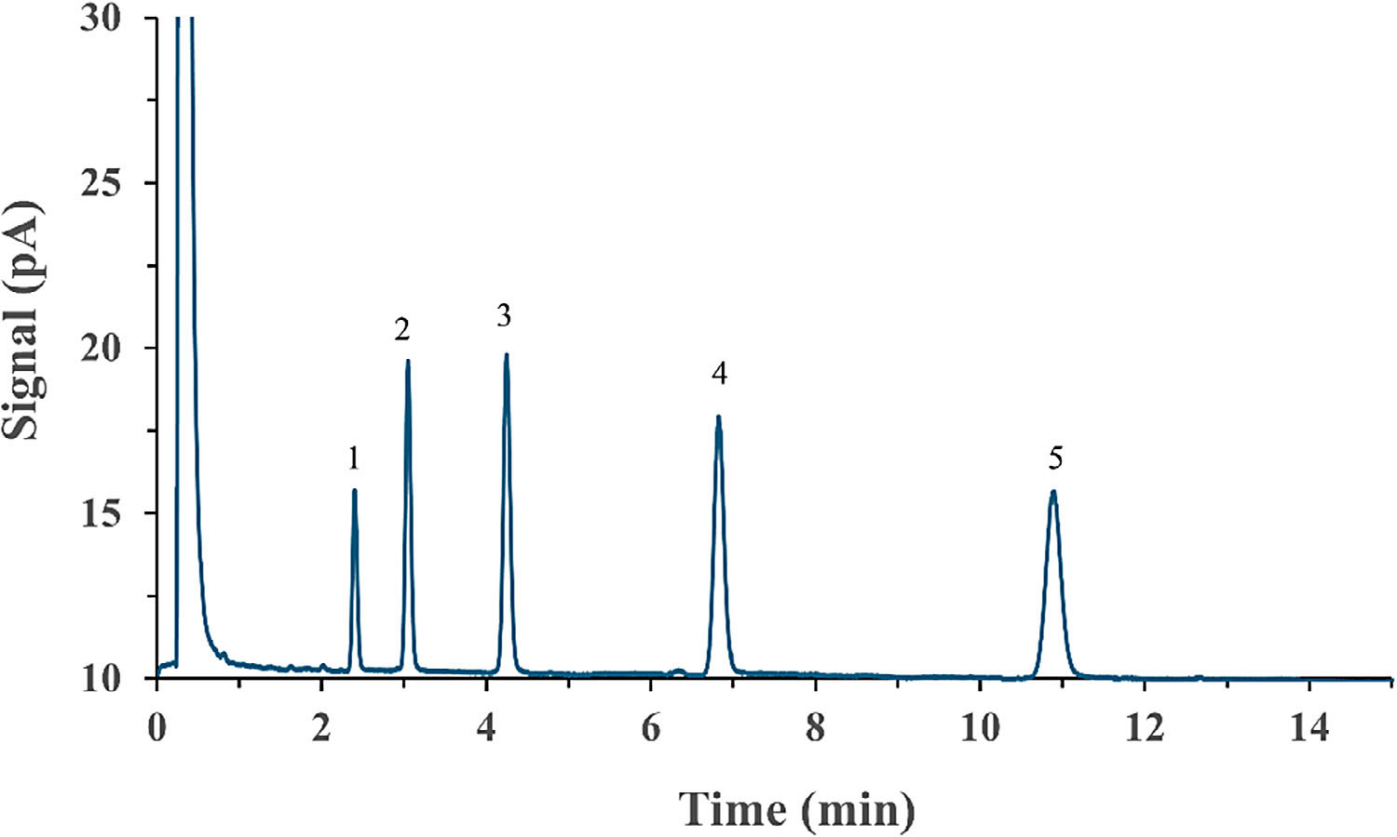
Chromatographic separation of five volatile carboxylic acids under isothermal conditions at 100°C: The analytes are: 1. acetic acid, 2. propionic acid, 3. butyric acid, 4. valeric acid, and 5. hexanoic acid. The column was coated with a mixture of acrylate zwitterionic liquid and 40% poly(ethylene glycol) methacrylate on an untreated capillary and subjected to polymerization and thermal conditioning prior to carrying out the separations.

To obtain a more uniform coating of the stationary phase, untreated fused silica capillary columns were used for these experiments. Different weight percentages of PEGMA (10, 20, 25, and 40 wt%) relative to the acrylate ZIL were investigated to evaluate the effect of incorporated co‐monomer on the rigidity of the polymerized stationary phase, with the resulting changes being assessed through chromatographic performance measurements. After polymerization and conditioning, the chromatographic performance of the columns was evaluated by using benzyl alcohol as a probe. The retention time of benzyl alcohol was observed to increase with PEGMA content up to 25% (48.0 ± 0.7, 50.1 ± 0.4, and 56.4 ± 0.4 min for 10, 20, and 25% PEGMA, respectively), followed by a slight decrease at 40% PEGMA (44.5 ± 0.1 min). Column efficiencies up to 3300 plates m^−^^1^ were observed for PEGMA‐containing columns, as shown in Table 1. The chromatographic performance of the stationary phases was further evaluated using VCAs. For this experiment, a column containing 40% PEGMA was selected as a representative composition to evaluate the separation capability of the modified stationary phase. The column demonstrated effective separation of the tested VCAs with good peak asymmetry values (<1.25 for all VCAs) as shown in Table 2 and Figure 4, indicating that incorporation of PEGMA did not compromise the chromatographic selectivity of the ZIL stationary phase. When compared to the cross‐bonded commercial Stabilwax column, the acrylate ZIL with 40% PEGMA exhibited higher retention for VCAs, as shown in Table 2. For example, the retention factors for acetic acid were 8.07 ± 0.03 and 3.729 ± 0.001, while those for hexanoic acid were 40.0 ± 0.3 and 30.72 ± 0.05 on the acrylate ZIL with 40% PEGMA and Stabilwax columns, respectively. In addition, the acrylate ZIL with 40% PEGMA provided lower peak asymmetry factors for VCAs (1.04–1.19) compared to Stabilwax (1.10–1.47).

**TABLE 2:** Retention factor and peak asymmetry factor at 100°C for five volatile carboxylic acids on a column coated with a mixture of acrylate zwitterionic liquid with 40% poly(ethylene glycol) methacrylate on an untreated capillary after polymerization and conditioning, as discussed in section 2.3, and with the commercial Stabilwax column after conditioning. All investigated columns were 5 m × 0.25 mm I.D.

	Acrylate ZIL with 40% PEGMA^a^		Stabilwax	
Volatile carboxylic acids	Retention factor^b^ ± SD	Peak asymmetry factor ± SD	Retention factor^b^ ± SD	Peak asymmetry factor ± SD
Acetic acid	8.07 ± 0.03	1.18 ± 0.01	3.729 ± 0.001	1.10 ±0.04
Propionic acid	10.5 ± 0.1	1.16 ± 0.02	5.924 ± 0.001	1.27 ± 0.05
Butyric acid	15.0 ± 0.1	1.19 ± 0.02	9.570 ± 0.002	1.47 ± 0.05
Valeric acid	24.7 ± 0.2	1.17 ± 0.05	17.286 ± 0.004	1.42 ± 0.01
Hexanoic acid	40.0 ± 0.3	1.04 ± 0.07	30.72 ± 0.05	1.16 ± 0.07

^a^ ZIL: zwitterionic liquid, PEGMA: poly(ethylene glycol) methacrylate.

^b^ Calculated using propane as a dead time marker.

SD: Standard deviation calculated with *n* = 3 measurements.

Following exposure of the columns to 250°C, chromatographic performance was reassessed at 100°C. During thermal conditioning, the bleed intensity of the PEGMA‐containing columns (i.e., 182.95 pA for 40% PEGMA containing column at 250°C) remained comparable to that observed for the polymerized ZIL column without PEGMA (154.67 pA at 250°C, as shown in Figure S12), indicating that incorporation of the co‐monomer did not adversely affect the thermal stability of the stationary phase. Under these conditions, progressively narrower peaks of benzyl alcohol were observed with increased PEGMA content (3.3 ± 0.2, 1.10 ± 0.01, 0.81 ± 0.01, and 0.194 ± 0.001 for 10, 20, 25, and 40% weight fractions, respectively, as shown in Table S3 and Figure S13), suggesting that incorporation of PEGMA reduced the rigidity of the polymerized stationary phase and improved stationary phase mass transfer. This behavior can be attributed to the presence of flexible PEG segments, which can increase chain mobility within the polymer network and make the stationary phase less restrictive to analyte diffusion [28]. The retention time of benzyl alcohol was found to decrease to approximately half of its initial value after high temperature exposure (12.02 ± 0.03 min before thermal degradation to 6.32 ± 0.01 min after thermal degradation), although separation efficiencies of up to 1200 plates m^−^^1^ were still obtained for the column containing 40% PEGMA. When separation efficiencies were measured using phenol, efficiencies of nearly 1500 plates m^−^^1^ could be achieved, as shown in Table S3.

To provide a comparison on the balance between PEGMA and ZIL content, a column containing 100% PEGMA without ZIL was also prepared and subjected to polymerization, followed by an evaluation of efficiency testing using benzyl alcohol and separation of VCAs (Figure S14). In this case, the chromatographic peaks exhibited noticeable tailing (PAFs > 1.64 for all VCAs), indicating that PEGMA alone does not provide favorable chromatographic interactions for these analytes. After exposure to 250°C, the 40% PEGMA/ZIL column showed co‐elution of acetic and propionic acids, whereas the 100% PEGMA column maintained separation of these compounds (Figure S15), highlighting the different interaction mechanisms provided by PEGMA and ZIL within the stationary phase. Despite coelution, the 40% PEGMA‐ZIL column showed improved peak asymmetry for valeric and hexanoic acid (1.9 ± 0.1 and 2.0 ± 0.1, respectively) and longer retention for all VCAs (for example, 2.54 ± 0.02 min for acetic acid and 6.20 ± 0.01 min for hexanoic acid) than the 100% PEGMA column (PAFs of 2.74 ± 0.09 and 3.8 ± 0.7 for valeric and hexanoic acid, respectively; retention times of 0.804 ± 0.002 min for acetic acid and 4.10 ± 0.01 min for hexanoic acid), indicating that the ZIL component primarily contributes to favorable interactions with the VCAs.

These results indicate that PEGMA effectively reduces the rigidity of the polymerized ZIL phase. Despite these improvements, the overall kinetic performance of the columns remained modest. The highest efficiency observed following thermal conditioning was approximately 1500 plates m^−^^1^ for phenol, which remains substantially lower than efficiencies typically achieved with commercial GC stationary phases. In addition, the decrease in benzyl alcohol retention following exposure to 250°C and the co‐elution of acetic and propionic acids on the 40% PEGMA/ZIL column indicate that thermal exposure can alter stationary phase properties in ways that negatively affect chromatographic performance. These observations suggest that although polymerization successfully reduced column bleed, increased network rigidity, and restricted analyte diffusion likely continue to limit mass transfer within the stationary phase. Despite these limitations, the successful preparation of a low‐bleed polymerized ZIL stationary phase demonstrates the potential of this approach and provides a foundation for further optimization. Future studies should therefore explore alternative classes of co‐monomers and polymer architectures to further improve column efficiency and maintain effective VCA separation after exposure to elevated temperatures.

## 4 Conclusions

In this study, polymerizable imidazolium‐based ZILs were investigated as stationary phases in the development of thermally stable GC phases, maintaining the ability to separate and discriminate between VCAs. Of the evaluated chemical structures, only the acrylate ZIL demonstrated the ability to undergo polymerization. Following in‐situ polymerization and achieving chromatographic efficiencies up to 3300 plates m^−1^, the stationary phase could separate VCAs under isothermal conditions at 100°C and exhibited reduced bleed (from 6.92–7.80 pA at 40°C to 154.70–157.57 pA at 250°C) compared to an analogous non‐polymerizable ZIL (from 7.57 pA at 40°C to 396.38 pA at 250°C), indicating improved thermal stability of the stationary phase upon polymerization. However, exposure of the columns to 250°C resulted in elution of the efficiency probe with the solvent, suggesting increased rigidity of the polymerized phase. Incorporation of PEGMA as a co‐monomer reduced this effect and improved the stationary phase mass transfer (as observed by decreasing peak widths of 3.3 ± 0.2, 1.10 ± 0.01, 0.81 ± 0.01, and 0.194 ± 0.001 min for 10, 20, 25, and 40% weight fractions, respectively) after high temperature exposure.

Future work should focus on evaluating alternative co‐monomers to better balance polymer rigidity and chromatographic performance after thermal exposure. In addition, identifying suitable surface modification strategies for GC capillaries that allow effective bonding of the polymerizable moieties may further improve column efficiency and chromatographic performance.

## Author Contributions

**Bhawana Thapa**: experimental design; conceptualization; writing – original draft; literature review. **Jessica F. DeLair**: experimental design; conceptualization; writing – original draft; literature review. **Jared L. Anderson**: supervision, writing – review and editing, project administration and funding.

## Conflicts of Interest

The authors declare no conflicts of interest.

## Declaration of Artificial Intelligence‐assisted Technologies

The authors did not use artificial intelligence‐assisted technologies in the creation of figures/tables or in the interpretation of data.

## Supporting information




**Supporting File**: jssc70496‐sup‐0001‐SuppMat.docx.

## Data Availability

The data supporting this study's findings are available from the corresponding author upon reasonable request.
