# Supplementary material for: Polymerizable Zwitterionic Liquid Stationary Phases With Improved Thermal Stability for Separation of Carboxylic Acids
Source: J Sep Sci. 2026 Jul 22;49(7):e70496. doi: 10.1002/jssc.70496 (PMC13389833; doi:10.1002/jssc.70496)
Supplement: Supplementary file 1 — Supporting File: jssc70496‐sup‐0001‐SuppMat.docx. [file JSSC-49-e70496-s001.docx]

**Supporting Material**

**Polymerizable Zwitterionic Liquid Stationary Phases with Improved Thermal Stability for Separation of Carboxylic Acids**

Bhawana Thapa^†^, Jessica F. DeLair^†^, Jared L. Anderson*

*Department of Chemistry, Iowa State University, Ames, Iowa 50011 USA*

* Corresponding Author. *E-mail address:* andersoj@iastate.edu (J. L. Anderson)

^†^ These authors contributed equally to this work.

**Table of Contents:**

**Synthesis of 3-(3-(2-(2-(vinyloxy)ethoxy)ethyl)-1*H*-imidazol-3-ium-1-yl)propane-1-sulfonate (VinylPEG-2ImC_3_SO_3_) zwitterionic liquid ~~……~~……………….………………………Page S3**

**Synthesis of 3-(1-(oct-7-en-1-yl)-1*H*-imidazol-3-ium-3-yl)propane-1-sulfonate (OctenylImC_3_SO_3_) zwitterionic liquid ~~…….~~……………………………………………Page S5**

**Synthesis of 3-(1-(6-(acryloyloxy)hexyl)-1*H*-imidazol-3-ium-3-yl)propane-1-sulfonate (acrylate zwitterionic liquid)……………….……………………………………………Page S7**

**Synthesis of 3-(1-octyl-1*H*-imidazol-3-ium-3-yl)propane-1-sulfonate (C_8_ImC_3_SO_3_)……………………………………………………………………………...Page S9**

**Table S1……………………………………………………………………….…………Page S10**

**Table S2……………………………………………………………………….…………Page S11**

**Table S3……………………………………………………………………….…………Page S12**

**Figure S1…………………………………………………………………………………Page S13**

**Figure S2…………………………………………………………………………………Page S14**

**Figure S3…………………………………………………………………………………Page S15**

**Figure S4…………………………………………………………………………………Page S16**

**Figure S5…………………………………………………………………………………Page S17**

**Figure S6…………………………………………………………………………………Page S18**

**Figure S7……………………………………………………………………………........Page S19**

**Figure S8…………………………………………………………………………………Page S20**

**Figure S9…………………………………………………………………………………Page S21**

**Figure S10………………………………………………………………………………..Page S22**

**Figure S11………………………………………………………………………………..Page S23**

**Figure S12………………………………………………………………………………..Page S24**

**Figure S13………………………………………………………………………………..Page S25**

**Figure S14………………………………………………………………………………..Page S26**

**Figure S15………………………………………………………………………………..Page S27**

**Synthesis of 3-(3-(2-(2-(vinyloxy)ethoxy)ethyl)-1*H*-imidazol-3-ium-1-yl)propane-1-sulfonate (VinylPEG-2ImC_3_SO_3_)**

The synthesis of VinylPEG_2_ImC_3_SO_3_ was carried out through modification of a procedure reported in a previous study [1]. Di(ethylene glycol) vinyl ether (VinylPEG-2) was used in place of diethylene glycol monomethyl ether. For purification, HCl quenching was not employed, and the purification procedure was optimized as described below. The reaction scheme is shown in Fig. S1.

VinylPEG-2 (6.2 mmol) was dissolved in 5 mL of dichloromethane (DCM) and placed in an ice bath for 30 minutes. Approximately 2.6 mL of triethylamine (TEA) was measured out using a syringe and added to 10 mL of DCM. The resulting solution was added dropwise (1 drop per 5 s) to the cooled PEG solution. Following the addition of TEA, 6.2 mmol solution of methanesulfonyl chloride (MsCl) in 10 mL of DCM was subsequently added dropwise to the reaction solution. After the addition of MsCl, the reaction was allowed to return to room temperature and continued to react overnight. The crude mixture was subjected to six washings with DI water. After removing the solvent, the product was dried in a vacuum oven overnight. A final yield of 70.1 % was obtained. The resulting product was a clear, low viscosity liquid.

*Synthesis of 1-(2-(2-(vinyloxy)ethoxy)ethyl)-1H-imidazole (ImvinylPEG-2)*

Imidazole (4.7 mmol) was reacted with 9.5 mmol of KOH at room temperature for 30 minutes in 10 mL of acetonitrile. Ms-vinyl-PEG-2 (4.31 mmol) in 2 mL of acetonitrile was added and allowed to react for 3 days at room temperature, with the reaction progress monitored each day using ^1^H NMR. Once completed, the reaction solution was filtered using Fisher brand filter paper (P5, medium porosity). The reaction flask was rinsed twice with 20 mL of acetonitrile (ACN) and the rinses also subjected to filtration. ACN and residual water formed during the reaction was then removed via a rotary evaporator to dryness. The product was redissolved in DCM and washed 4 times with water to remove excess imidazole, and DCM was removed via rotary evaporation. Finally, the product was rinsed with ether to remove residual ions, and ether was removed using an air stream and the products were dried in a vacuum oven overnight. A final yield of 58.03 % was obtained.

*Synthesis of VinylPEG-2ImC_3_SO_3_ zwitterionic liquid*

Finally, 3.5 mmol of 1,3-propanesultone was reacted with 3.83 mmol of ImvinylPEG-2 in 8 mL of acetonitrile at 80 °C for 24 h until the reaction was complete. ACN was removed, and the crude product was dried overnight in a vacuum oven. The final product was purified by dissolving the product in water and washing five times with DCM. Water was then removed using rotary evaporation and further drying was performed by placing the product in a room temperature vacuum oven. The VinylPEG-2ImC_3_SO_3_ ZIL was a translucent, highly viscous liquid with a slight yellowish tint.

**Synthesis of 3-(1-(oct-7-en-1-yl)-1*H*-imidazol-3-ium-3-yl)propane-1-sulfonate (OctenylImC_3_SO_3_) zwitterionic liquid**

OctenylImC_3_SO_3_ was synthesized using a modified procedure from a previous study [1] *which is described as follows:*

*Synthesis of N-(2-cyanoethyl)imidazole (PrIm)*

To synthesize PrIm, 0.098 mmol of imidazole was added to a reflux system and purged with nitrogen gas to obtain an inert system. Then, 0.121 mmol of acrylonitrile was dissolved in 5 mL of methanol and added via syringe to the reflux system and allowed to react at 45 °C for 5 hours. Following reaction completion, methanol and excess acrylonitrile were removed by rotary evaporation. The PrIm product was then placed in a vacuum oven for 2 days.

*Synthesis of 1-(2-cyanoethyl)-3-(oct-7-en-1-yl)-1H-imidazol-3-ium bromide ([PrIm^+^Octenyl][Br^-^])*

The synthesis of the intermediate [PrIm^+^Octenyl][Br^-^] occurred by reacting 6.41 mmol of PrIm with 8.97 mmol of 8-bromo-1-octene in 4 mL of chloroform at 80 °C for 3 days. Once the reaction was completed, the solvent was removed under an air stream. The resulting product was dissolved in 20 mL of acetonitrile and washed via LLE with 10 mL of hexane 13 times. The acetonitrile layer was collected, and the acetonitrile was removed by rotary evaporation. The product was a viscous, yellow liquid with a final yield of 77.8%.

*Synthesis of 1-octenylimidazole*

The deprotection of [PrIm^+^Octenyl][Br^-^] occurred by reacting 3.98 mmol of [PrIm^+^Octenyl][Br^-^] with 7.7 mmol of 15% (w/v) sodium hydroxide solution in 18 mL of DI-water. The mixture was stirred at 200 rpm for 2 hours. After 2 hours, the reaction mixture was transferred to a separatory funnel, and 20 mL of hexane was added to the separatory funnel. Significant emulsion formed between the two layers but eventually dissipated. The hexane layer was then washed via LLE with 10 mL of DI-water until a silver nitrate test confirmed no salt by-products or starting materials were present. The aqueous layer appeared cloudy but gradually became clear after repeated washings. The hexane layer was collected, and hexane was removed via rotary evaporation. The final product was a low-viscosity yellow liquid with a final yield of 27%.

*Synthesis of OctenylImC_3_SO_3_*

The final product was synthesized by reacting 1.56 mmol of 1-octenylimidazole with 1.56 mmol of 1,3-propanesultone in 624 µL of acetonitrile at room temperature for 3 days. Following reaction completion, the solvent was removed under an air stream, and the crude product was rinsed 20 times with diethyl ether, using occasional sonication. The final product was then dried in a vacuum oven. The product OctenylImC_3_SO_3_ was a high-viscosity liquid with a slight yellow tint. The final yield was 91%.

**Synthesis of 3-(1-(6-(acryloyloxy)hexyl)-1*H*-imidazol-3-ium-3-yl)propane-1-sulfonate (acrylate zwitterionic liquid)**

Synthesis of the acrylate ZIL was developed as a two-step reaction as described below and the scheme is provided in Fig. S2.

*Synthesis of sodium 3-(1H-imidazol-1-yl)propane-1-sulfonate ([ImC_3_SO_3_^-^] [Na^+^])*

Deprotonation of imidazole was first carried out by reacting imidazole (11.4 mmol) with potassium hydroxide (22.8 mmol) in 4 mL of dimethyl sulfoxide (DMSO). The mixture was stirred at room temperature for 20 min. Subsequently, sodium 3-bromopropane-1-sulfonate (9.4 mmol) was dissolved in DMSO and added to the reaction mixture while keeping the total DMSO volume to a minimum at 16 mL. The resulting mixture was stirred at room temperature for 24 h to obtain [ImC_3_SO_3_^-^] [Na^+^]. Following completion of the reaction, excess KOH was removed by filtration, and the DMSO removed using rotary evaporation. The crude product was then purified using a methanol salt precipitation procedure. Briefly, the reaction mixture was suspended in methanol, centrifuged, and the supernatant was decanted. This process was repeated several times (until no white precipitate was observed) to facilitate removal of inorganic impurities. The resulting solid was then rinsed with acetone (5 mL, five times) to remove residual DMSO. Further purification was carried out by dissolving the product in water and washing with ethyl acetate (15X) to remove excess imidazole. Following removal of water using rotary evaporation, the product was subsequently rinsed with hot ethanol to eliminate residual salts and ions and was finally dried. The final product was a sticky brown solid or highly viscous liquid with a yield of 79.7 %.

*Synthesis of acrylate zwitterionic liquid*

[ImC_3_SO_3_^-^] [Na^+^] (2.4) mmol was reacted with 3.3 mmol of 6-bromohexyl acrylate in 4 mL of dimethylformamide (DMF) at 40 ºC for 5 days. After completion of the reaction, DMF was removed using rotary evaporation. The crude product was then dissolved in ACN and washed with hexane (20X) to remove excess 6-bromohexyl acrylate. ACN was then removed under rotary evaporation and the product further dried by placing it in vacuum oven overnight. The product was then stored in a desiccator by covering it with aluminum foil. A final yield of 62.8 % was obtained.

**Synthesis of 3-(1-octyl-1*H*-imidazol-3-ium-3-yl)propane-1-sulfonate (C_8_ImC_3_SO_3_) zwitterionic liquid**

The ZIL C_8_ImC_3_SO_3_ was synthesized following a previously reported method by Zeger et al. [1] in which 5.4 mmol of 1-octylimidazole was reacted with 5.4 mmol of 1,3-propanesultone at room temperature for 48 hours. The resulting product was purified by rinsing 20 times with 4 mL of diethyl ether. The final yield was 83.4%.

**Reference:**

1. V.R. Zeger, B. Thapa, J.F. DeLair, D.S. Bell, D. Shollenberger, M. Chakraborty, J.S. Herrington, J.L. Anderson, “Thermally stable and polar zwitterionic liquid stationary phases for gas chromatography: Understanding the impact of chemical structure,” Journal of Chromatography A 1740 (2025): 465594, https://doi.org/10.1016/j.chroma.2024.465594.

**Table S1.** Retention factor and peak asymmetry factor at 100 ºC for four volatile carboxylic acids on chromatographic column coated with 100 % acrylate zwitterionic liquid on untreated capillary after polymerization and conditioning, as described in section 2.3, on investigated 5 m × 0.25 mm I.D. columns.

| Volatile carboxylic acids | Retention factor ^a^ ± SD | Peak asymmetry factor ± SD |
| --- | --- | --- |
| Propionic acid | 11.62 ± 0.03 | 1.30 ± 0.03 |
| Butanoic acid | 15.1 ± 0.1 | 1.16 ± 0.02 |
| Valeric acid | 22.5 ± 0.2 | 1.07 ± 0.06 |
| Hexanoic acid | 32.6 ± 0.1 | 0.87 ± 0.06 |

^a^ Calculated using propane as a dead time marker.

SD: standard deviation calculated with n =3 measurements

**Table S2.** Retention factor and peak asymmetry factor at 100 ºC for four volatile carboxylic acids on a chromatographic column coated with 100 % acrylate zwitterionic liquid on vinyl trimethoxy silane modified capillary after polymerization and conditioning as described in section 2.3, on investigated 5 m × 0.25 mm I.D. columns.

| Volatile carboxylic acids | Retention factor ^a^ ± SD | | Peak asymmetry factor ± SD | Peak width at half height ± SD |
| --- | --- | --- | --- | --- |
| Propionic acid | 7.9 ± 0.1 | 1.33 ± 0.03 | | 0.437 ± 0.003 |
| Butanoic acid | 10.09 ± 0.04 | 1.38 ± 0.03 | | 0.582 ± 0.013 |
| Valeric acid | 14.53 ± 0.04 | 1.39 ± 0.02 | | 0.839 ± 0.009 |
| Hexanoic acid | 20.7 ± 0.2 | 1.34 ± 0.05 | | 1.123 ± 0.005 |

^a^ Calculated using propane as a dead time marker.

SD: standard deviation calculated with n =3 measurements

**Table S3.** Retention factor, column efficiency and peak width of benzyl alcohol at 100 ºC after column exposure to 250 ºC, as described in section 2.3, on investigated 5 m × 0.25 mm I.D. columns.

| Stationary phase | Retention factor ^a^ ± SD | | Chromatographic efficiency (plates m^-1^)^b^ ± SD | Peak width at half height |
| --- | --- | --- | --- | --- |
| Acrylate ZIL + 10 % PEGMA | 26.5 ± 0.1 | n.d. | | 3.3 ± 0.2 |
| Acrylate ZIL + 20 % PEGMA | 27.6 ± 0.1 | 50 ± 1 | | 1.10 ± 0.01 |
| Acrylate ZIL + 25 % PEGMA | 29.4 ± 0.1 | 105 ± 2 | | 0.81 ± 0.01 |
| Acrylate ZIL + 40 % PEGMA | 22.9 ± 0.1 | 1170 ± 6, 1450 ± 40 ^c^ | | 0.194 ± 0.001 |
| 100 % PEGMA | 17.72 ± 0.01 | 750 ± 20 | | 0.189 ± 0.001 |
| Stabilwax | 33.05 ± 0.03 | 3220 ± 30 | | 0.1633 ± 0.0007 |

^a^ Calculated using propane as a dead time marker

^b^ Determined using the width at half-height of the chromatographic peak (benzyl alcohol)

^c^ Using phenol as probe molecule

n.d.: not determined.

SD: Standard deviation calculated with n =3 measurements.

**
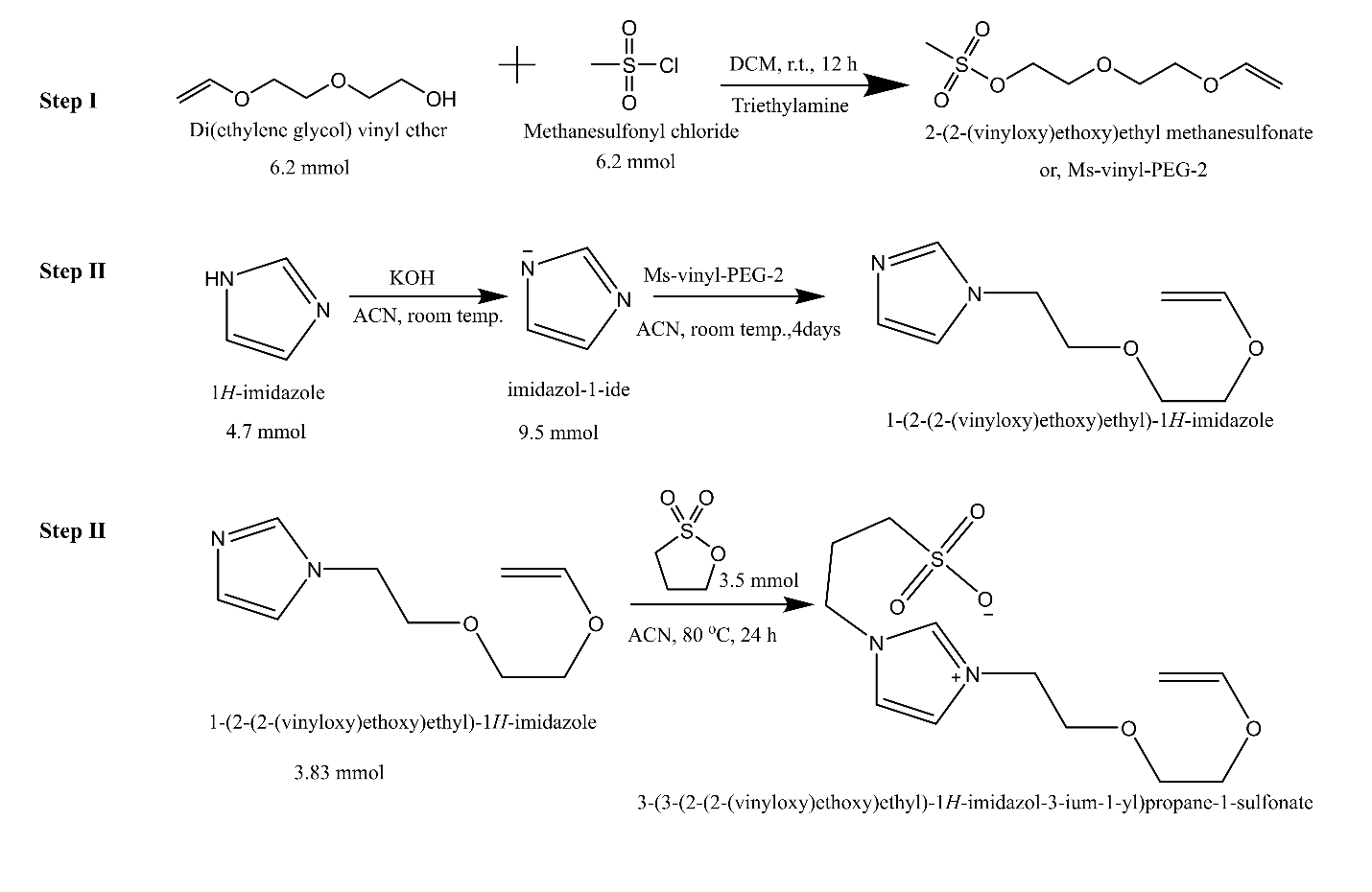
Figure S1.** Synthetic scheme used in the preparation of Vinyl-PEG-2ImC_3_SO_3_ zwitterionic liquid.


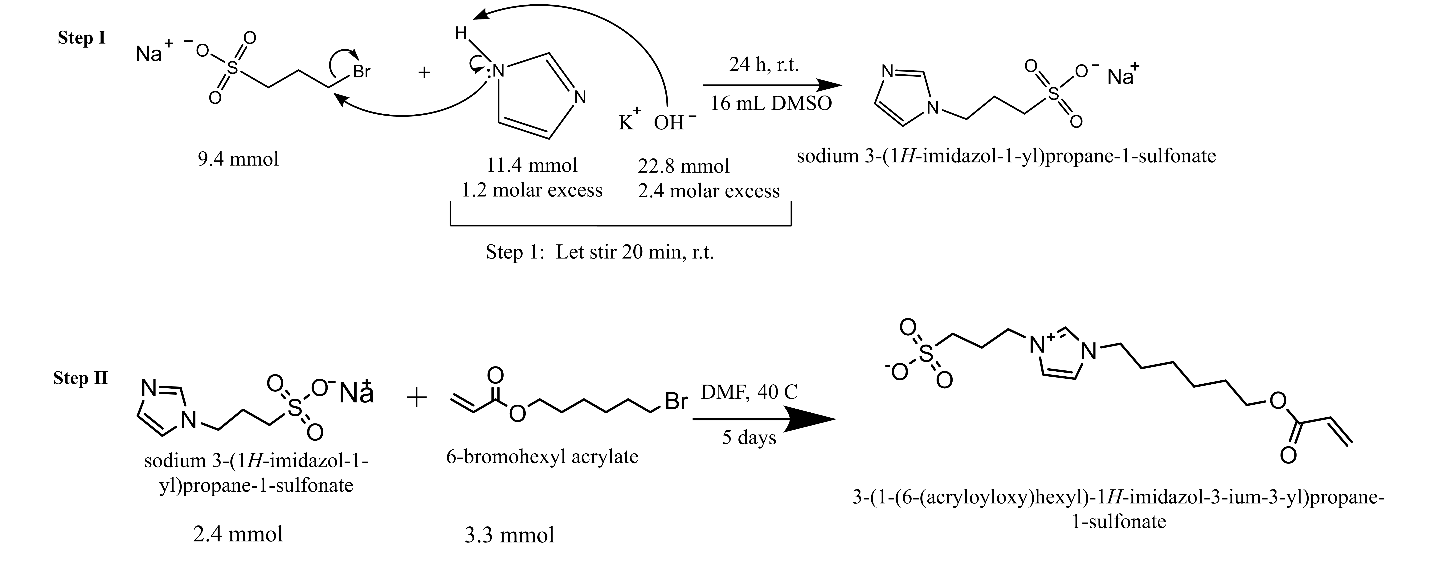


**Figure S2.** Synthetic scheme of the acrylate zwitterionic liquid.

**
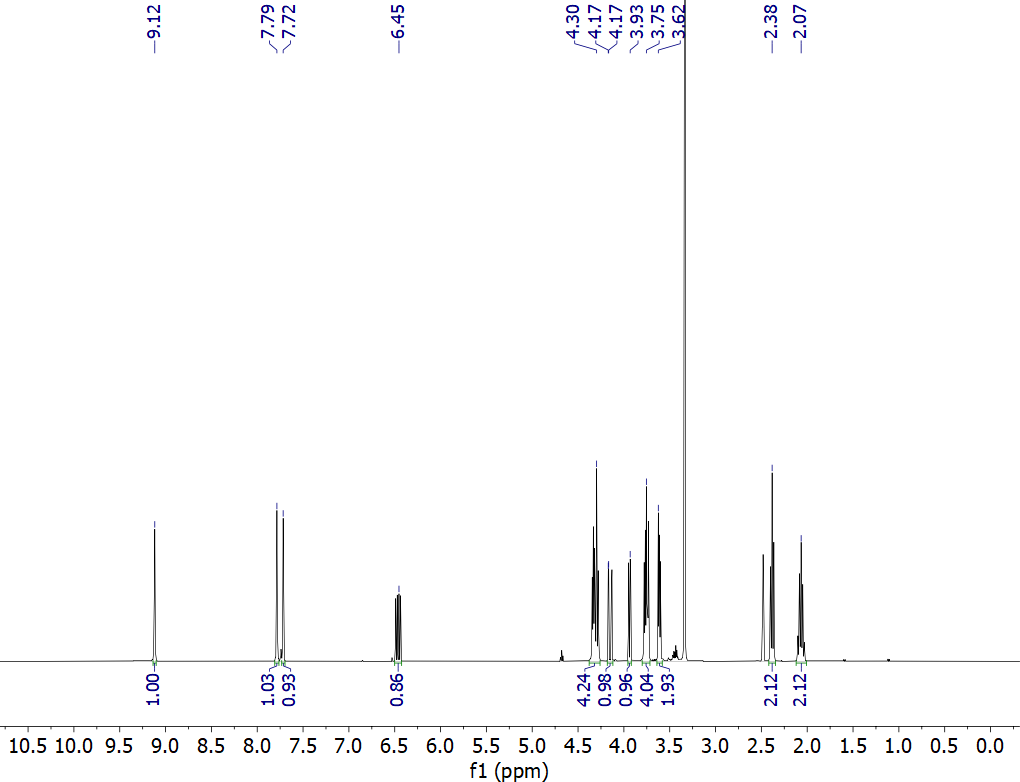
Figure S3.** Proton NMR spectrum of VinylPEG-2ImC_3_SO_3_. ^1^H NMR (400 MHz, DMSO) δ 9.12 (s, 1H), 7.79 (s, 1H), 7.72 (s, 1H), 6.45 (dd, J = 14.3, 6.7 Hz, 1H), 4.37 – 4.24 (m, 4H), 4.17 (dd, J = 14.3, 1.8 Hz, 1H), 3.93 (dd, J = 6.7, 1.8 Hz, 1H), 3.75 (ddd, J = 8.8, 5.0, 3.4 Hz, 4H), 3.65 – 3.58 (m, 2H), 2.38 (dd, J = 7.7, 6.7 Hz, 2H), 2.07 (p, J = 7.1 Hz, 2H). Impurities peaks are indicated by *.

*

*

*

*

**
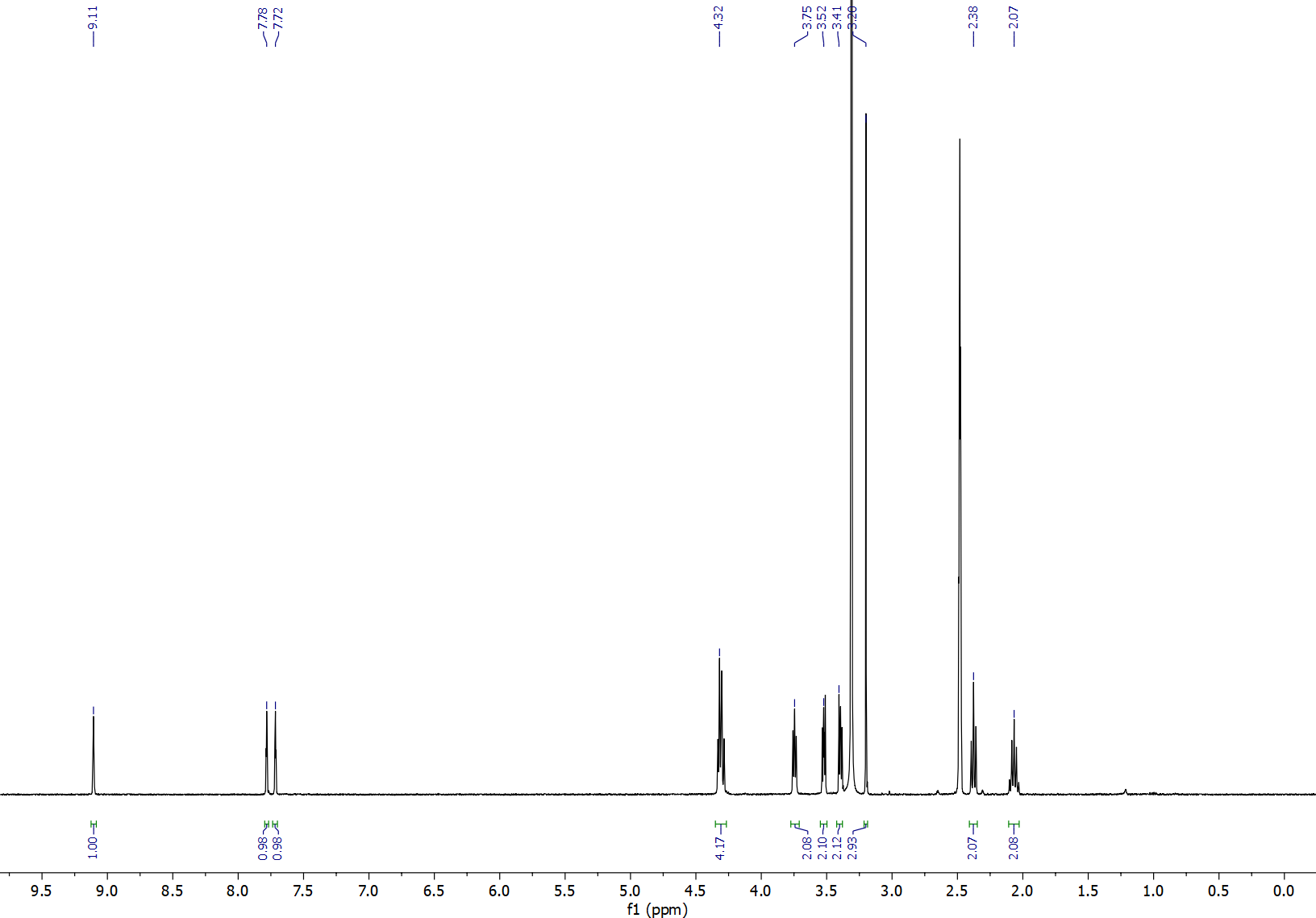
Figure S4.** Proton NMR spectrum showing loss of vinyl groups and appearance of a new peak at 3.21 ppm upon storing VinylPEG-2ImC_3_SO_3_ in desiccator. ^1^H NMR (400 MHz, DMSO) δ 9.12 (s, 1H), 7.80 (s, 1H), 7.73 (s, 1H), 4.35 – 4.24 (m, 4H), 3.75 (dd, J = 5.5, 4.4 Hz, 2H), 3.5 – 3.49 (m, 2H), 3.43 – 3.34 (m, 2H), 3.20 (s, 3H), 2.38 (dd, J = 7.7, 6.7 Hz, 2H), 2.07 (p, J = 7.1 Hz, 2H).


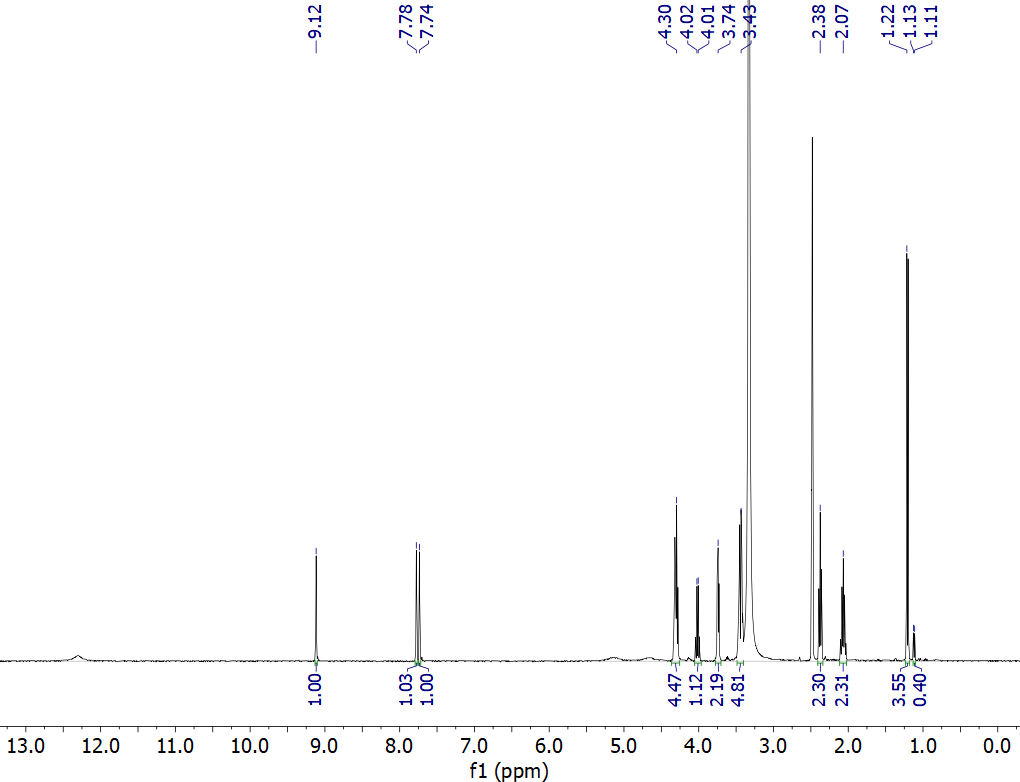
**Figure S5.** Proton NMR spectrum showing loss of vinyl groups and appearance of new peaks at 1.2 and 1.1 ppm upon storing VinylPEG-2ImC_3_SO_3_ in a room temperature vacuum oven overnight. ^1^H NMR (400 MHz, DMSO) δ 9.12 (s, 1H), 7.76 (dt, J = 16.5, 1.8 Hz, 2H), 4.35 – 4.26 (m, 4H), 4.02 (q, J = 6.9 Hz, 1H), 3.78 – 3.71 (m, 2H), 3.50 – 3.39 (m, 4H), 2.38 (t, J = 7.2 Hz, 2H), 2.07 (p, J = 7.0 Hz, 2H), 1.21 (d, J = 6.9 Hz, 3H), 1.13 (dd, J = 8.5, 5.2 Hz, 1H).

**Figure S6.** Proton NMR spectrum for 3-(1-(oct-7-en-1-yl)-1*H*-imidazol-3-ium-3-yl)propane-1-sulfonate. ^1^H NMR (400 MHz, DMSO) δ 9.18 (s, 1H), 7.79 (d, *J* = 7.0 Hz, 2H), 5.79 (d, *J* = 6.8 Hz, 1H), 5.05 – 4.89 (m, 2H), 4.30 (s, 2H), 4.15 (s, 2H), 2.39 (s, 2H), 2.07 (s, 2H), 2.01 (q, *J* = 6.9 Hz, 2H), 1.79 (s, 2H), 1.29 (d, *J* = 27.9 Hz, 6H).

**
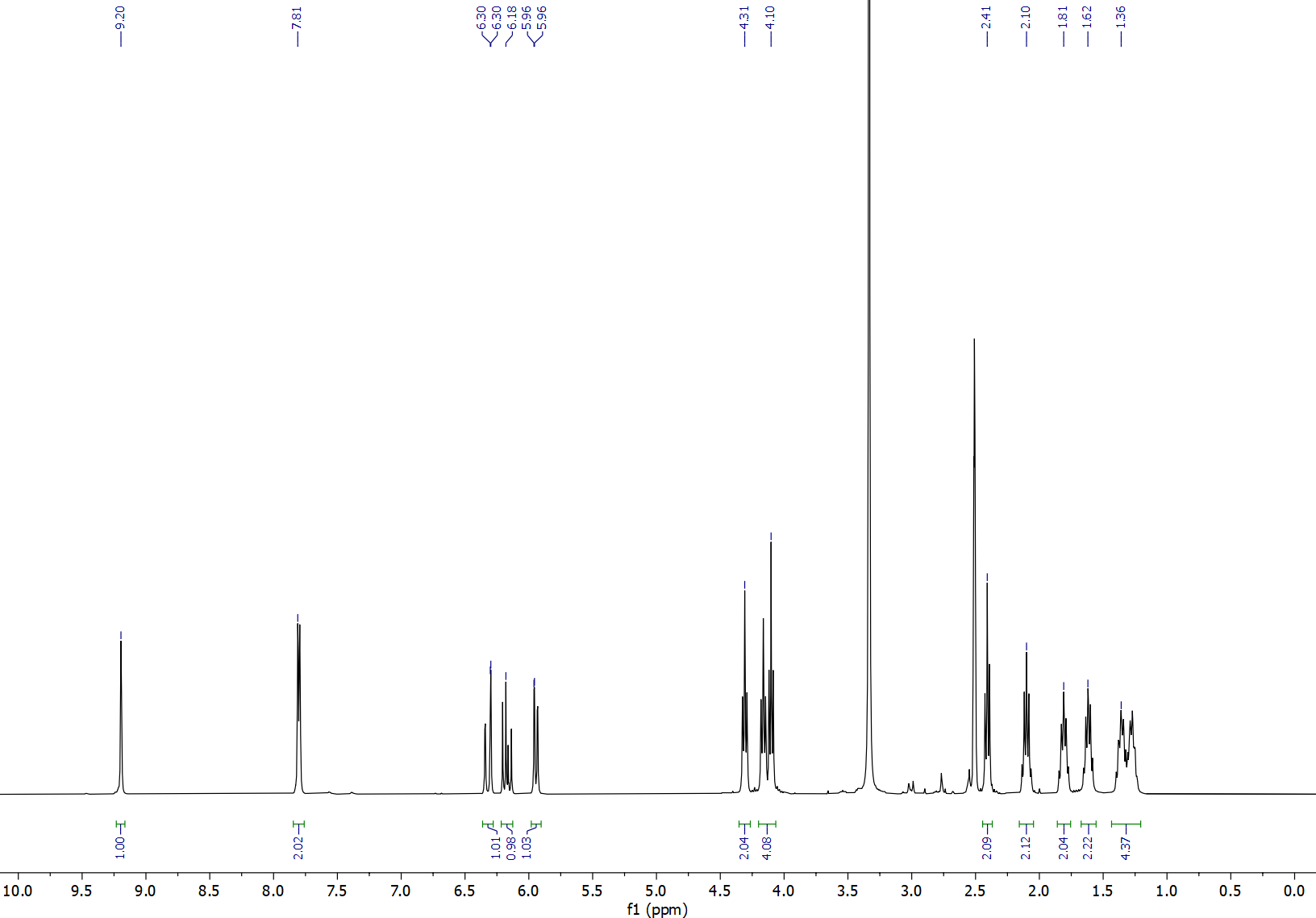
Figure S7.** Proton NMR spectrum of acrylate zwitterionic liquid. ^1^H NMR (400 MHz, DMSO) δ 9.20 (s, 1H), 7.78 (dt, J = 6.6, 1.8 Hz, 2H), 6.30 (dd, J = 17.3, 1.6 Hz, 1H), 6.14 (dd, J = 17.3, 10.3 Hz, 1H), 5.93 (dd, J = 10.3, 1.7 Hz, 1H), 4.31 (t, J = 7.0 Hz, 2H), 4.16 (dt, J = 23.5, 6.9 Hz, 4H), 2.41 (t, J = 7.2 Hz, 2H), 2.13 – 2.01 (m, 2H), 2.10 (m, 2H), 1.81 (p, J = 7.4 Hz, 2H), 1.62 (p, J = 6.7 Hz, 2H), 1.36 (ddt, J = 36.4, 14.7, 6.9 Hz, 4H).

**
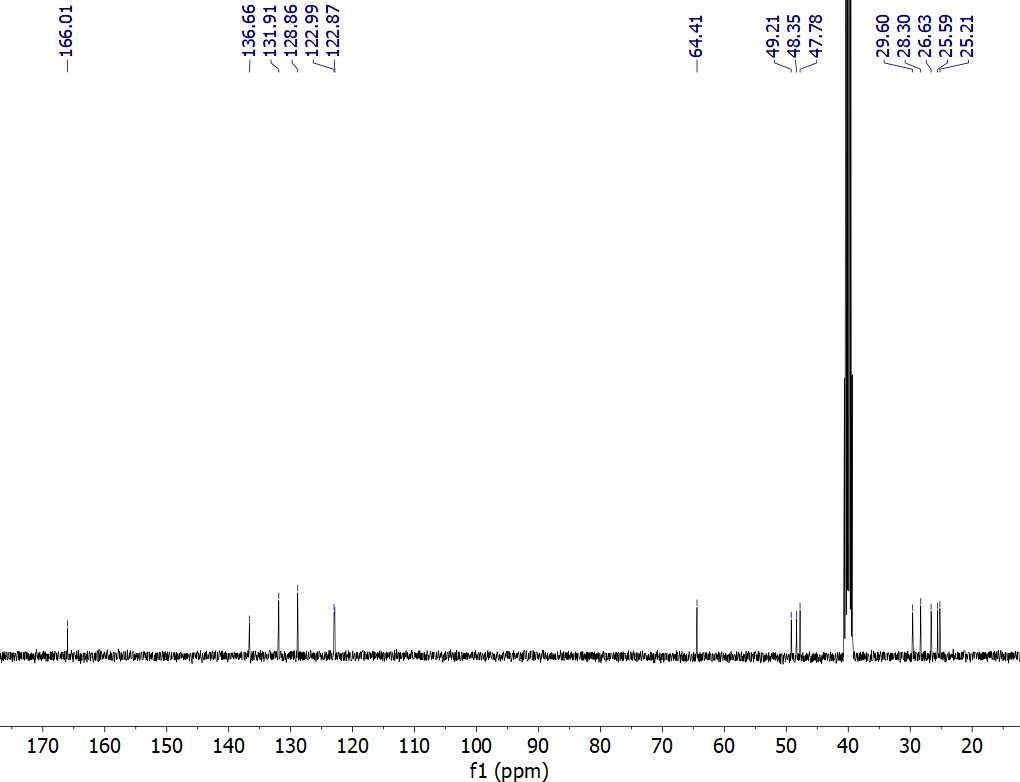
Figure S8.** ^13^C NMR spectrum of acrylate zwitterionic liquid. ^13^C NMR (101 MHz, DMSO) δ(ppm) 166.01, 136.66, 131.91, 128.86, 122.99, 122.87, 64.41, 49.21, 48.35, 47.78, 29.60, 28.30, 26.63, 25.59, 25.21.

**
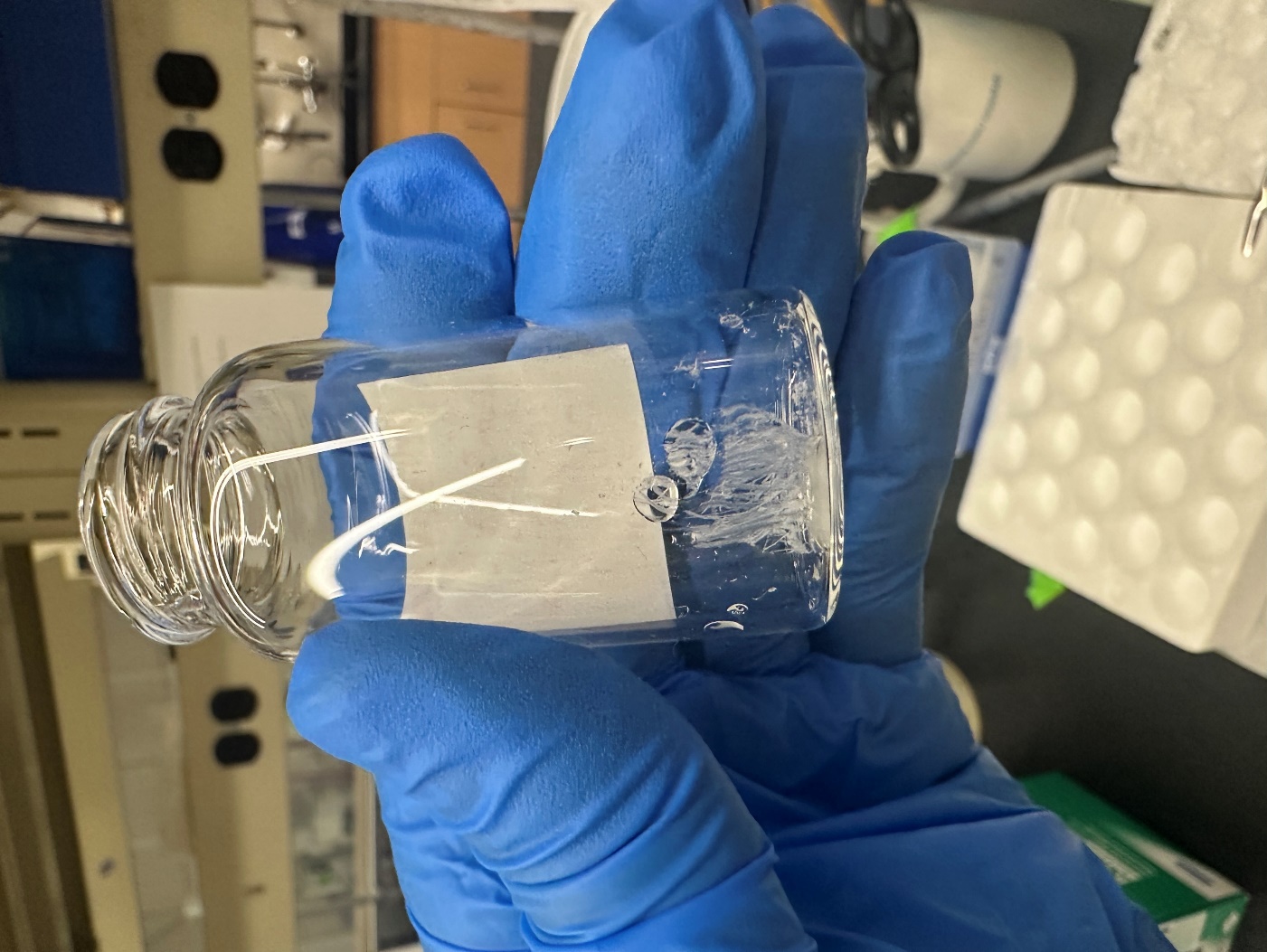
Figure S9.** Photograph of the rigid solid polymer formed upon heating the acrylate zwitterionic liquid inside the vacuum oven at 60 ºC overnight.

**
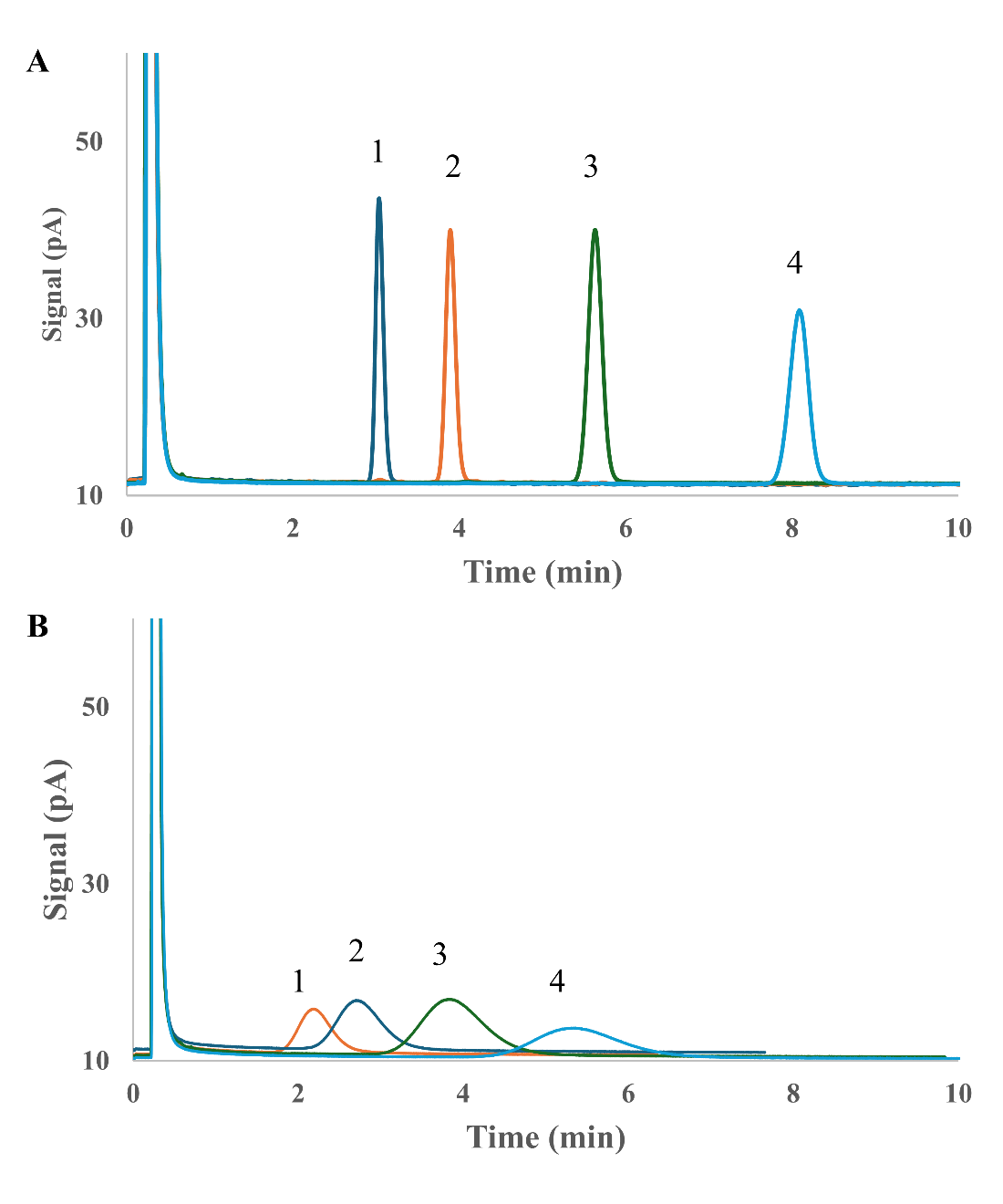
Figure S10.** Overlayed chromatograms showing separation of the following four volatile carboxylic acids: 1. propionic acid, 2. butanoic acid, 3. valeric acid, 4. hexanoic acid on chromatographic columns coated with acrylate zwitterionic liquid on untreated (A) and vinyl trimethoxy silane modified capillary (B). Separations were carried out isothermally at 100 ºC.

**
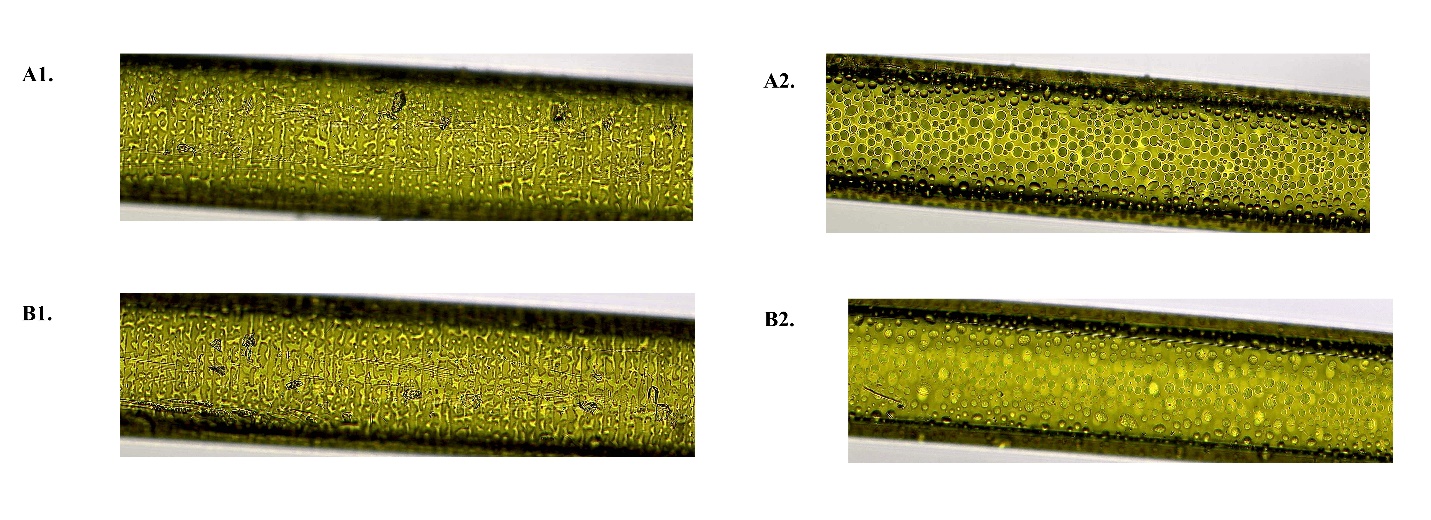
Figure S11.** Optical microscopy images of the acrylate zwitterionic liquid stationary phase coated on vinyl trimethoxy silane modified capillary after polymerization, (A1.) vacuum end and (A2.) sealed end and after exposure to 250 ºC, B1. vacuum end and B2. sealed end.

**
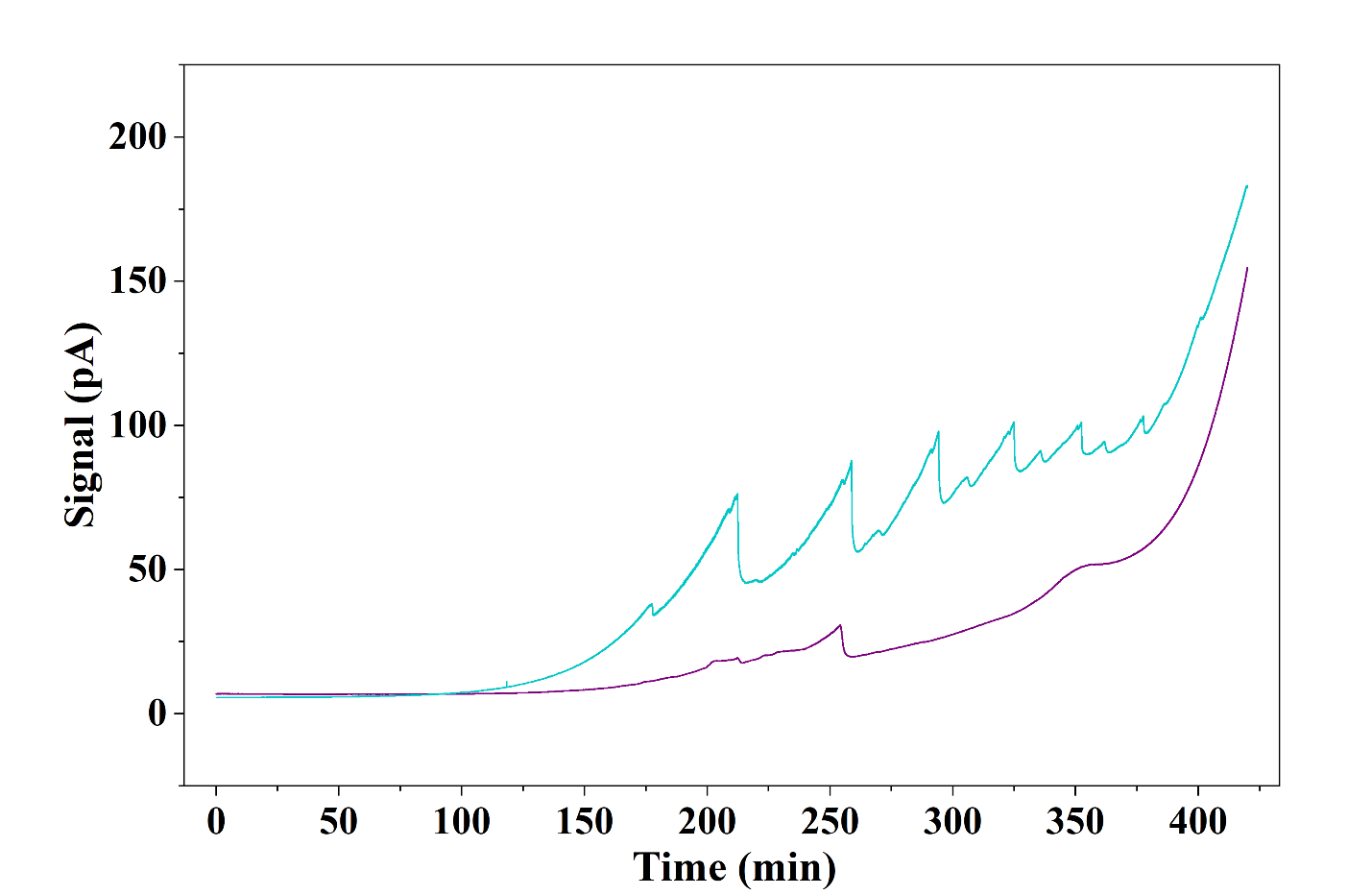
Figure S12.** Bleed profile of columns coated with 100 % acrylate zwitterionic liquid (purple), and mixture of zwitterionic liquid with 40 % poly(ethylene glycol) methacrylate (blue) on untreated capillary. Bleed studies were conducted using the following temperature program: 40 to 250 °C at 0.5 °C min^-1^.

**
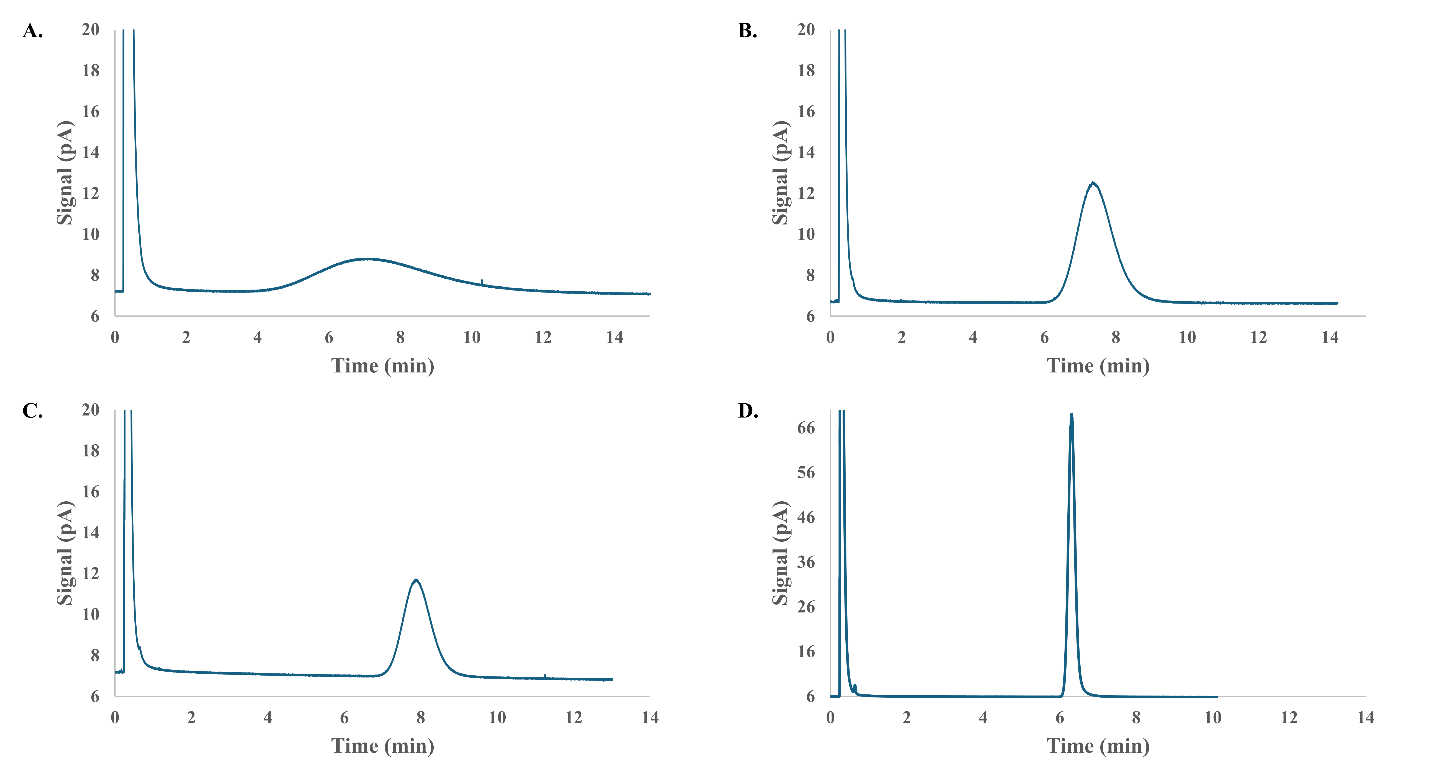
Figure S13.** Chromatograms showing progressive improvement in peak width of benzyl alcohol upon increasing the content of poly(ethylene glycol) methacrylate; (A.) 10 %, (B.) 20 %, (C.) 25 % and (D.) 40 %. Benzyl alcohol was injected at 100 ºC after exposing all the columns to 250 ºC. Separations were carried out isothermally at 100 ºC.

**
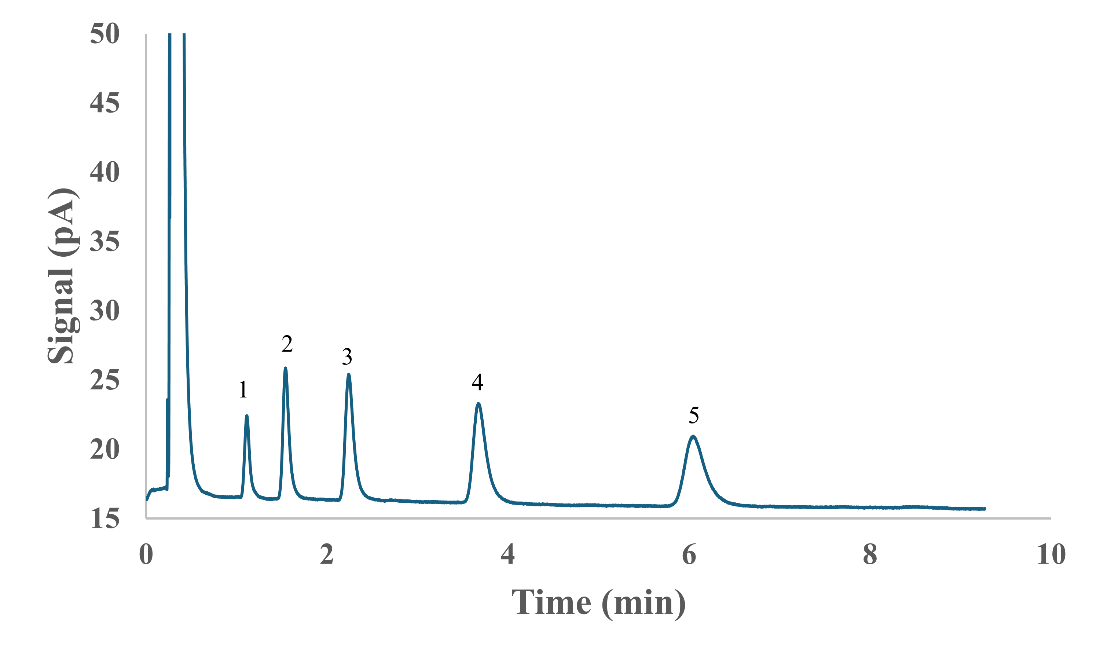
Figure S14.** Chromatographic separation of five volatile carboxylic acids: 1. acetic acid, 2. propionic acid, 3. butanoic acid, 4. valeric acid and 5. hexanoic acid on a column coated with 100 % poly(ethylene glycol) methacrylate stationary phase on untreated capillary after polymerization and conditioning. Separations were carried out isothermally at 100 ºC.

**
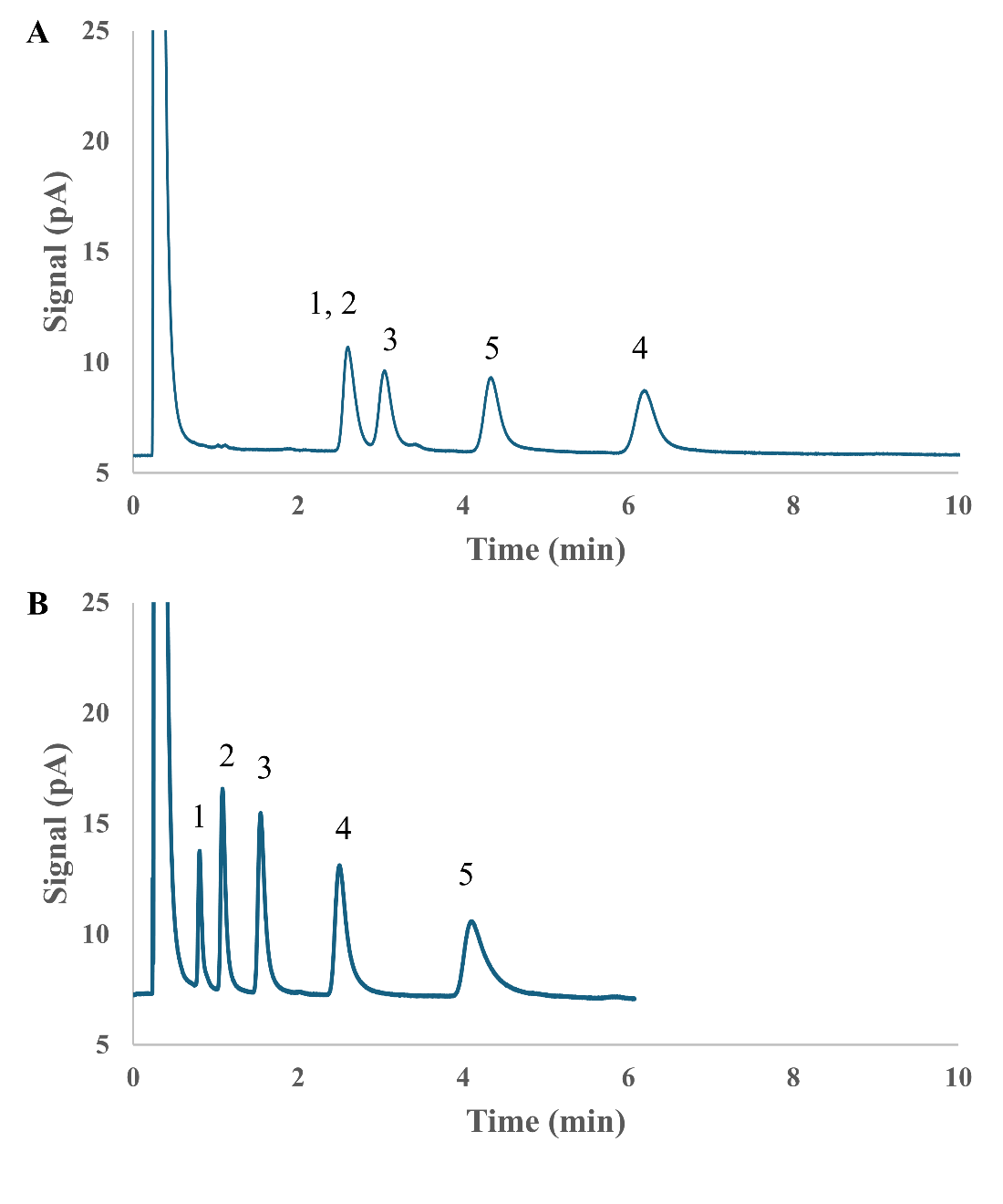
Figure S15.** Chromatographic separation of five volatile carboxylic acids: 1. acetic acid, 2. propionic acid, 3. butanoic acid, 4. valeric acid and 5. hexanoic acid on untreated capillary coated with (A.) mixture of zwitterionic liquid and 40 % poly(ethylene glycol) methacrylate and (B.) 100 % poly(ethylene glycol) methacrylate post exposure to 250 ºC. Separations were carried out isothermally at 100 ºC.
